# Optimized Synthesis and Characterization of Janus RhSeCl with Uniform Anionic Valences, Nonlinear Optical and Optoelectronic Properties

**DOI:** 10.1002/advs.202505279

**Published:** 2025-06-20

**Authors:** Kefeng Liu, Xuelian Sun, Puxin Cheng, Zhiteng Li, Penghui Li, Donghan Jia, Shijing Zhao, Xin Yang, Xinyu Wang, Liangting Ye, Shengqing Xia, Shuo Zhang, Yu Chen, Tao Gan, Jiong Li, Xiao Zhang, Jialiang Xu, Anmin Nie, Bing Huang, Huiyang Gou

**Affiliations:** ^1^ Center for High Pressure Science and Technology Advanced Research (HPSTAR) Beijing 100094 P. R. China; ^2^ Beijing Computational Science Research Center Beijing 100193 P. R. China; ^3^ School of Materials Science and Engineering Tianjin Key Laboratory of Metal and Molecular Materials Chemistry Frontiers Science Center for New Organic Matter Nankai University Tianjin 300350 P. R. China; ^4^ State Key Laboratory of Information Photonics and Optical Communications School of Physical Science and Technology Beijing University of Posts and Telecommunications Beijing 100876 P. R. China; ^5^ Center for High Pressure Science State Key Laboratory of Metastable Materials Science and Technology Yanshan University Qinhuangdao 066044 P. R. China; ^6^ State Key Laboratory of Crystal Materials Institute of Crystal Materials Shandong University Shandong 250100 P. R. China; ^7^ Shanghai Synchrotron Radiation Facility Shanghai Advanced Research Institute Chinese Academy of Sciences Shanghai 201204 P. R. China; ^8^ Department of Physics Beijing Normal University Beijing 100875 P. R. China

**Keywords:** janus materials, nonlinear optical properties, optoelectronic properties, steric hindrance, uniform anionic valences

## Abstract

The symmetry‐breaking nature of Janus materials enables the design of multifunctional compounds with distinct properties that are inaccessible to traditional materials. However, the limited availability of intrinsically stable Janus materials hinders a complete understanding of their full potential. Here, the first millimeter‐sized Janus material, RhSeCl, is successfully synthesized through the precisely controlled chemical vapor transport (CVT) method. Single‐crystal X‐ray diffraction and high‐resolution transmission electron microscopy analyses reveal the Janus character of RhSeCl, emphasizing its strong correlation with steric hindrance. X‐ray absorption near‐edge structure (XANES) analyses demonstrate the highly unusual oxidation states of Rh and Se, underlining their critical role in determining the formation of the inherent Janus structure. Interestingly, a clear second‐harmonic generation (SHG) is observed in RhSeCl, weakening with the temperature. DFT calculations attribut the strong SHG response to the band nesting effect, with an intensity modulated by the temperature‐dependent interlayer coupling. Notably, its damage threshold surpassed that of Janus MoSSe. Furthermore, devices based on RhSeCl exhibit promising optoelectronic performance at the visible wavelength range of 405–650 nm, showing a great opportunity for solar applications. These findings deepen the understanding of inherent Janus structures, paving the way for the development of new Janus compounds with versatile functionalities in advanced materials.

## Graphical abstract

Janus RhSeCl exhibits a facial heteroatom configuration and uniform anionic valences with Se and Cl in the −1 oxidation state, stabilizing the unique structure. The symmetry‐breaking nature enables promising nonlinear optical and optoelectronic performance, providing new insights into two–dimensional (2D) Janus materials and broadening their potential for advanced functional applications.

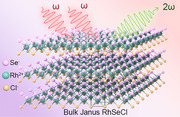

## 1 Introduction

Janus materials represent a unique subset of 2D compounds with asymmetric structure and composition. The asymmetry stems from selectively functionalizing or modifying one side of the material while leaving the other side unchanged or differently altered. This design imparts remarkable properties, such as tunable electronic structures,^[^ ^1^, ^2^ ^]^ enhanced catalytic activity,^[^ ^3^, ^4^ ^]^ and unique optical properties.^[^ ^5^, ^6^ ^]^ A prominent example is Janus graphene, where functionalizing just one side of the graphene sheet opens new possibilities for various applications.^[^ ^7^, ^8^ ^]^ For example, hydrogenating or fluorinating one side of graphene introduces a bandgap, converting it from a semimetal to a semiconductor, which holds promise for diverse electronic applications.^[^ ^1^, ^7^, ^9^, ^10^ ^]^ Similarly, Janus transition metal chalcogenides, such as MoSSe and WSSe, exhibit an asymmetry in their chalcogen atoms that induces a built‐in electric field, enhancing their spintronic and catalytic properties.^[^ ^11^, ^12^ ^]^ Additionally, uniaxial strain in multilayer MoSTe results in significant out‐of‐plane piezoelectric polarization, comparable to that of traditional 3D piezoelectric materials.^[^ ^13^ ^]^ DFT calculations reveal that Janus 2D WSSe possesses a combination of desirable physical properties, including efficient charge carrier separation, favorable band edge potentials, and strong optical absorption in the visible spectrum, making it a promising candidate for next‐generation optoelectronic devices.^[^ ^14^, ^15^ ^]^

In recent years, Janus metal chalcohalides, such as BiTeI and RhSeCl, have emerged as a new frontier in this field. These materials feature both chalcogen and halogen atoms on opposite sides of the atomic layer, resulting in enhanced dipole moments and reactivity. This configuration makes them highly versatile for applications in ferroelectrics, piezoelectrics, and catalysis. For example, BiTeI exhibits a giant Rashba splitting effect, making it a promising candidate for future spintronic devices.^[^ ^16^ ^]^ Similarly, CrSCl displays the coexistence of piezoelectricity and ferromagnetism.^[^ ^17^ ^]^ Additionally, theoretical studies predict that these Janus materials may also exhibit nonlinear optical properties,^[^ ^18^, ^19^ ^]^ high photocatalytic efficiency,^[^ ^20^ ^]^ and improved thermoelectric performance.^[^ ^21^, ^22^ ^]^ Despite their potential, the development of Janus materials faces significant challenges. Their limited availability and the complexity of designing and synthesizing them pose substantial obstacles. Achieving precise control over atomic arrangement and composition on each side of the Janus structure, particularly at the bulk scale, remains difficult. The inherent asymmetry of these materials further complicates their structural stability and scalability. Additionally, challenges such as fluctuations in oxidation states, unforeseen phase transitions, and the intricate relationship between structural asymmetry and functionality need to be thoroughly addressed. Overcoming these challenges is crucial for unlocking the full potential of Janus materials in a wide range of applications.

In this study, through optimized synthetic conditions, high‐quality 2D Janus RhSeCl materials were successfully synthesized using solid‐state methods and chemical vapor transport, resulting in single crystals with dimensions reaching up to 1 mm. The crystal structure, characterized by heteroleptic cation‐centered layers, was meticulously investigated through single‐crystal XRD and HAADF‐STEM. XAS provided crucial insights into the valences and coordination environments of the constituents, deepening our understanding of the phase stability and chemical bonding of Janus material. Notably, second‐harmonic generation (SHG) signals were clearly observed in experiments for RhSeCl, with a noticeable decrease in intensity as the temperature increased. First‐principles calculations reveal that the enhanced SHG response is driven by the band nesting effect, while the temperature‐induced reduction in intensity is linked to a decrease in interlayer coupling strength. In addition, RhSeCl was also found to exhibit the optoelectronic performance at the wavelength range of 405–650 nm. These findings highlight the promise of Janus RhSeCl for advanced applications and encourage further research in nonlinear optics and material science.

## 2 Results and Discussion

### 2.1 Synthesis and Structural Feature of RhSeCl

**Figure 1:**
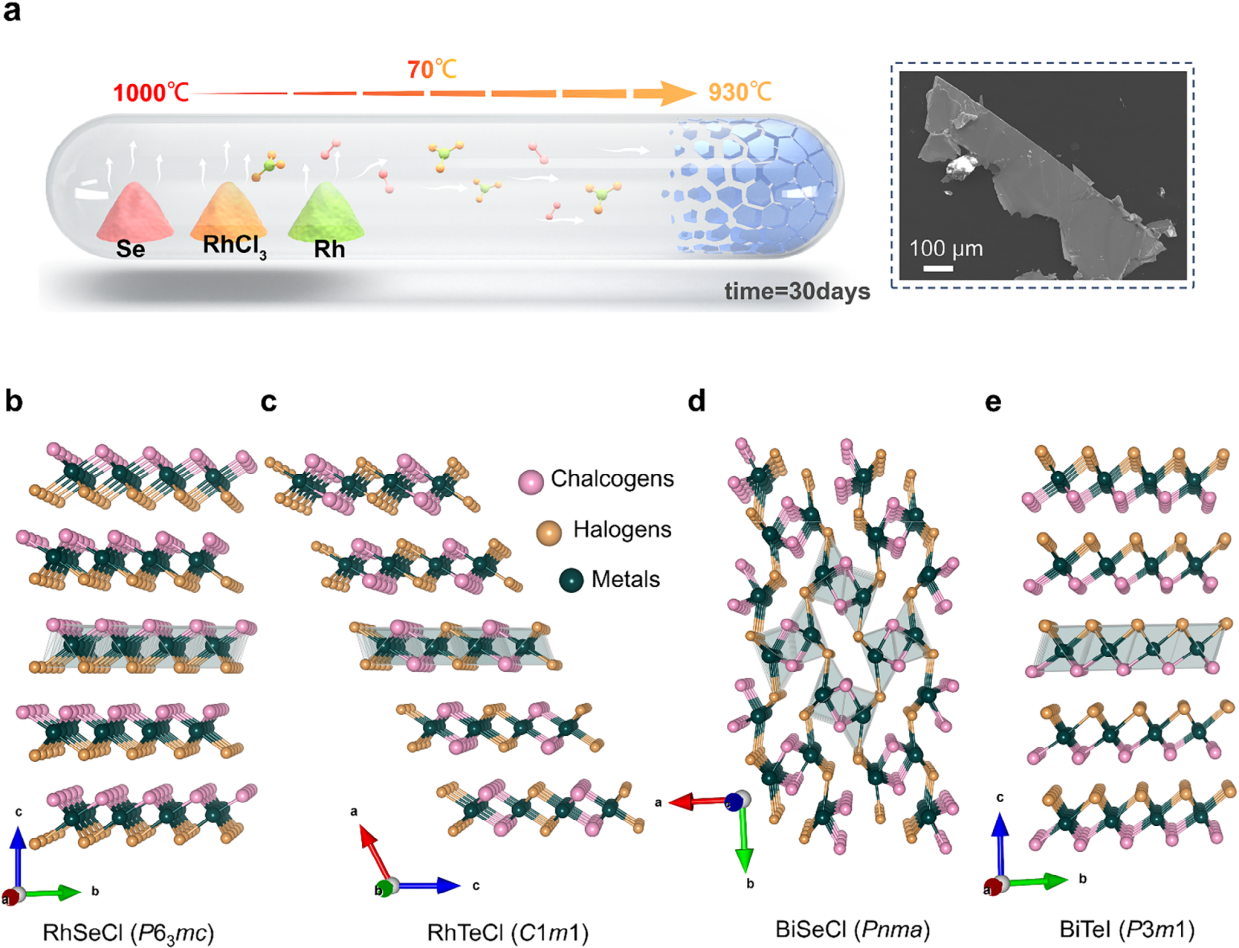
a) Optimized crystal growth process by the CVT method and SEM image of the as‐prepared crystals. Structural comparison of Janus‐related halogen‐bearing compounds, including b) RhSeCl, c) RhTeCl, d) BiSeCl, and e) BiTeI.

The synthesis of large single crystals of RhSeCl is inherently challenging due to inhomogeneities, which are common in Janus compounds. The significant difference between the melting point of Rh (1964 °C) and the sublimation temperature of Se (685 °C), along with issues related to the controllability and safety of chlorine sources, further complicates the process. Initial attempts using chemical vapor transport (CVT) only yielded small crystals <50 µm (Δ*T* = 100 K, *T*1 = 850 °C, *T*2 = 950 °C, and 5 days), as reported previously.^[^ ^23^ ^]^ To overcome these challenges, we develop an optimized synthetic approach with precise control over the process (**Figure** **1**a). We also introduced 10% RhCl_3_ to enhance the transport rate, which contributed to larger crystal growth.^[^ ^23^ ^]^ We find that crystal size is strongly dependent on the growth temperature. By increasing the source temperature to 1000 °C, sufficient activation energy is supplied, and we are able to effectively activate the reactants and accelerate the reaction rate. Additionally, we optimize the temperature gradient to a narrow Δ*T* of 70 K and set the sink temperature to 930 °C, which reduces thermal stress and defects, thereby facilitating the large and high‐quality crystals. Extending the dwelling time also plays a crucial role in enhancing the size of the crystals. These adjustments enable the successful synthesis of millimeter‐sized RhSeCl crystals (see Figure 1a), indicating the importance of process optimization in achieving large crystal growth.

RhSeCl crystallizes in a non‐centrosymmetric structure (space group *P*6_3_ *mc*, No. 186) at room temperature, with lattice parameters *a* = 3.4901(2) Å and *c* = 11.5941(7) Å, consistent with previous studies.^[^ ^23^ ^]^ This asymmetry is preserved over the temperature range of 30–500 K, with the *a*‐axis length remaining constant, while the *c*‐axis length increases from 11.549(2) Å to 11.652(1) Å (Figure S1, Supporting Information). Detailed structural parameters are summarized in Tables S1–S5 (Supporting Information). Rietveld refinement of the powder X‐ray diffraction pattern corroborates the single crystal analysis (Figure S2, Supporting Information). The structure features 2D RhSeCl blocking layers arranged in an ABAB sequence (Figure 1b). In each layer, each Rh atom is coordinated to three equivalent Se atoms and three Cl atoms, forming a Rh‐centered octahedron with bond lengths of 2.40 Å for Rh─Se and 2.52 Å for Rh─Cl, with adjacent octahedra sharing edges. The anion ordering in RhSeCl leads to a reduced Rh─Se bond length by ≈0.1 Å compared to other Rh─Se compounds,^[^ ^24^ ^]^ but results in an increased Rh─Cl bond length, ≈0.2 Å longer than that in RhCl_3_ with single anions.^[^ ^25^ ^]^

To explore the key structural factors that determine the formation of Janus structures, we compare several Janus and related compounds. RhTeCl features a 2D layered monoclinic structure where the larger Te atom decreases lattice symmetry (Figure 1c).^[^ ^26^ ^]^ While RhTeCl exhibits anion ordering, its bonding differs from that of RhSeCl. In RhSeCl, Se and Cl are connected on the same edge of the octahedron, whereas in RhTeCl, edges link to either the Te or Cl face, facilitating 2D networks of heteroatoms and reducing structural stress. As a result, milder synthetic conditions can be applied to RhTeCl, allowing crystal sizes to reach up to the hundred‐micron scale, with a sink temperature of 700 °C and shorter growth times.^[^ ^27^ ^]^ In contrast, BiTeCl also possesses a Janus structure^[^ ^28^ ^]^ with similar asymmetry to RhSeCl. Geometrically, anions serve as a matrix for cation formation, with cations acting as spatial fillers to maintain framework stability. Cation‐anion compatibility is thus a key structural consideration. Bi, with a larger covalent radius of 1.50 Å, is better suited for the coordination environment created by Te and Cl in the Janus system. However, incorporating Bi into Se and X (X═Cl, Br, I) frameworks leads to atomic size mismatches and lattice distortions, which break the symmetry of the Janus structure and adopt a 3D orthorhombic structure, as seen in BiSeX (Figure 1d). Furthermore, Janus BiTeBr and BiTeI with *P*3*m*1 symmetry (Figure 1e) exhibit greater angle distortions (∠Te‐Bi‐Br and ∠Te‐Bi‐I angles of 178.1° and 174.2°, respectively) compared to BiTeCl (178.5°). The degrees of octahedral distortion can be quantitatively evaluated using the formulas $\;\lambda_{oct}=\frac{1}{6}\;\sum_{i=1}^{6}{\left[\left(d_{i}-d_{0}\right)/d_{0}\right]}^{2}\;\mathrm{and}\;\delta_{oct}^{2}=\frac{1}{11}\;\sum_{i=1}^{12}{\left(\theta_{i}-90^{\circ }\right)}^{2}$, where *d_i_* and *θ_i_* represent individual bond lengths and angles, respectively, and *d_0_* is the average cation‐anion bond length.^[^ ^29^ ^]^ RhSeCl with the *λ_oct_* and *δ* ^2^ *_oct_* values of 5.95 × 10^−4^ and 4.15 deg^2^ shows smaller distortion than BiTeI with *λ_oct_* and *δ* ^2^ *_oct_* values of 1.46×10^−2^ and 13.47 deg^2^, highlighting size effects as a crucial factor for designing and synthesizing new Janus compounds in the future. The comparisons here suggest that when a metal's heteroleptic coordination number exceeds four, the metal‐halogen distances increase significantly (Table S6, Supporting Information), highlighting the role of steric hindrance in stabilizing the Janus system by mitigating repulsive interactions between the size‐mismatched anions (Se and Cl) and optimizing the spatial configuration around the central Rh atoms. In contrast to RhSeCl, Bi‐containing Janus compounds can be synthesized under less stringent reaction conditions, primarily due to the lower melting point and higher chemical reactivity of Bi.

**Figure 2:**
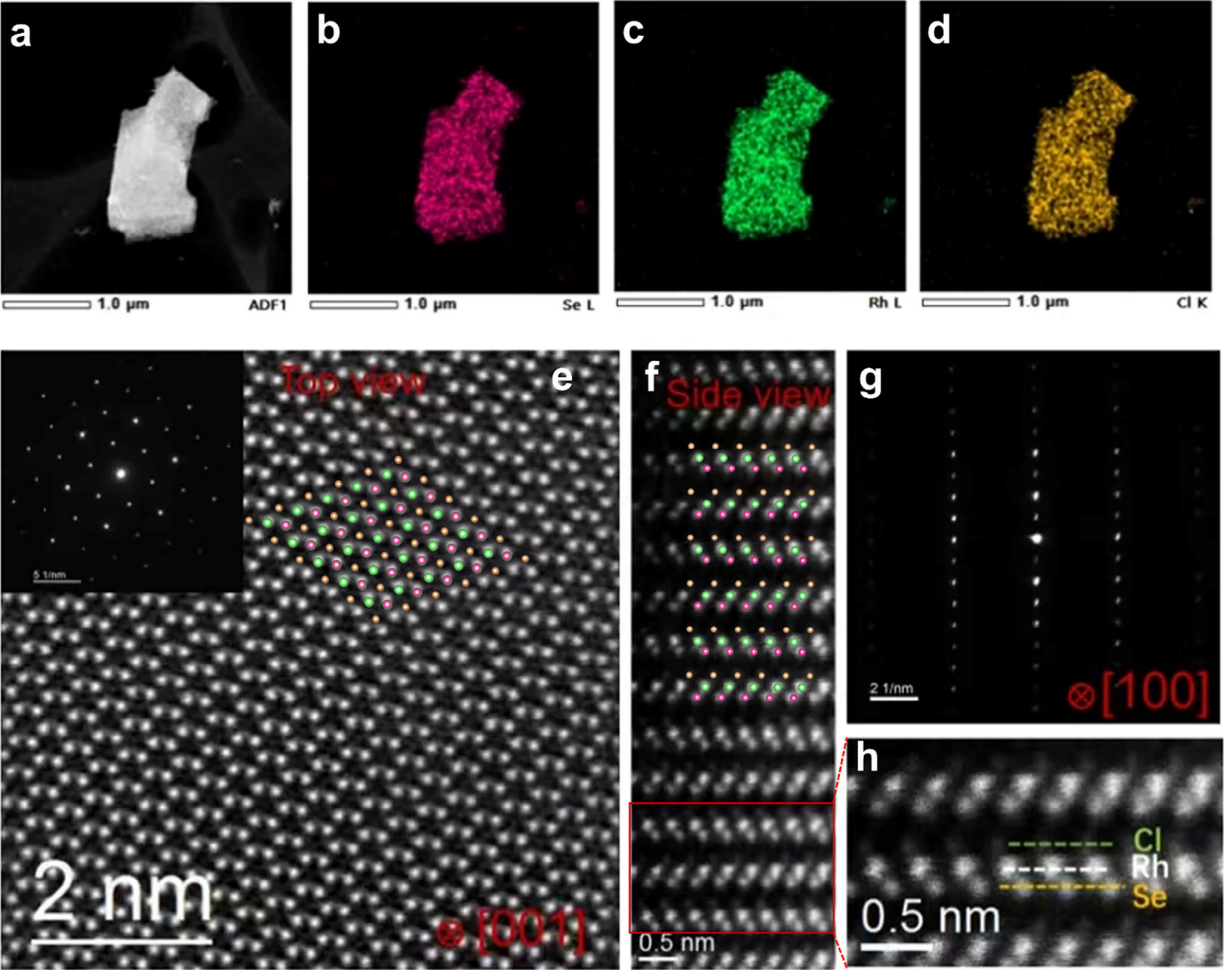
a–d) Low‐magnification images and simultaneously obtained STEM‐EDX elemental mappings of Rh L(green), Se L(pink), Cl K(orange). e) Atomic resolution HAADF‐STEM images of RhSeCl along the [001] zone axis with the corresponding SAED pattern inset. f) Atomic resolution HAADF‐STEM images of RhSeCl along the [100] zone axis, with the corresponding SAED pattern presented in g). h) The magnified image of the red rectangle box in f) is for clear illustration.

Scanning electron microscopy (SEM) images reveal the hexagonal symmetry in the crystallites, exhibiting distinct layered features (Figure 1a; Figure S3, Supporting Information) with the maximum size up to 1 mm. Energy dispersive X‐ray spectroscopy (EDX) confirms that the elemental composition with Rh:Se:Cl = 34.7(3):33.4(1):31.9(3), consistent with previously reported values.^[^ ^23^ ^]^ EDX mapping further demonstrates a uniform distribution of the component elements (Figure S4, Supporting Information), while scanning transmission electron microscopy with EDX (STEM‐EDX) mapping corroborates the chemical homogeneity of RhSeCl (**Figure** **2**a–d). The selected area electron diffraction (SAED) patterns, shown in the inset of Figure 2e, display six fold symmetry along the [001] zone axis and confirm the [100] axis orientation (Figure 2g). High‐resolution high‐angle annular dark‐field scanning transmission electron microscopy (HAADF‐STEM) images provide direct visualization of the atomic stacking. Cross‐sectional views along the (001) and (100) planes reveal a strong correlation between the atomic arrangements in the STEM images and their corresponding schematics in Figure 2e,f. These observations validate the Janus RhSeCl structure, with heteroatoms arranged in a facial configuration.

### 2.2 Chemical Feature of RhSeCl

**Figure 3:**
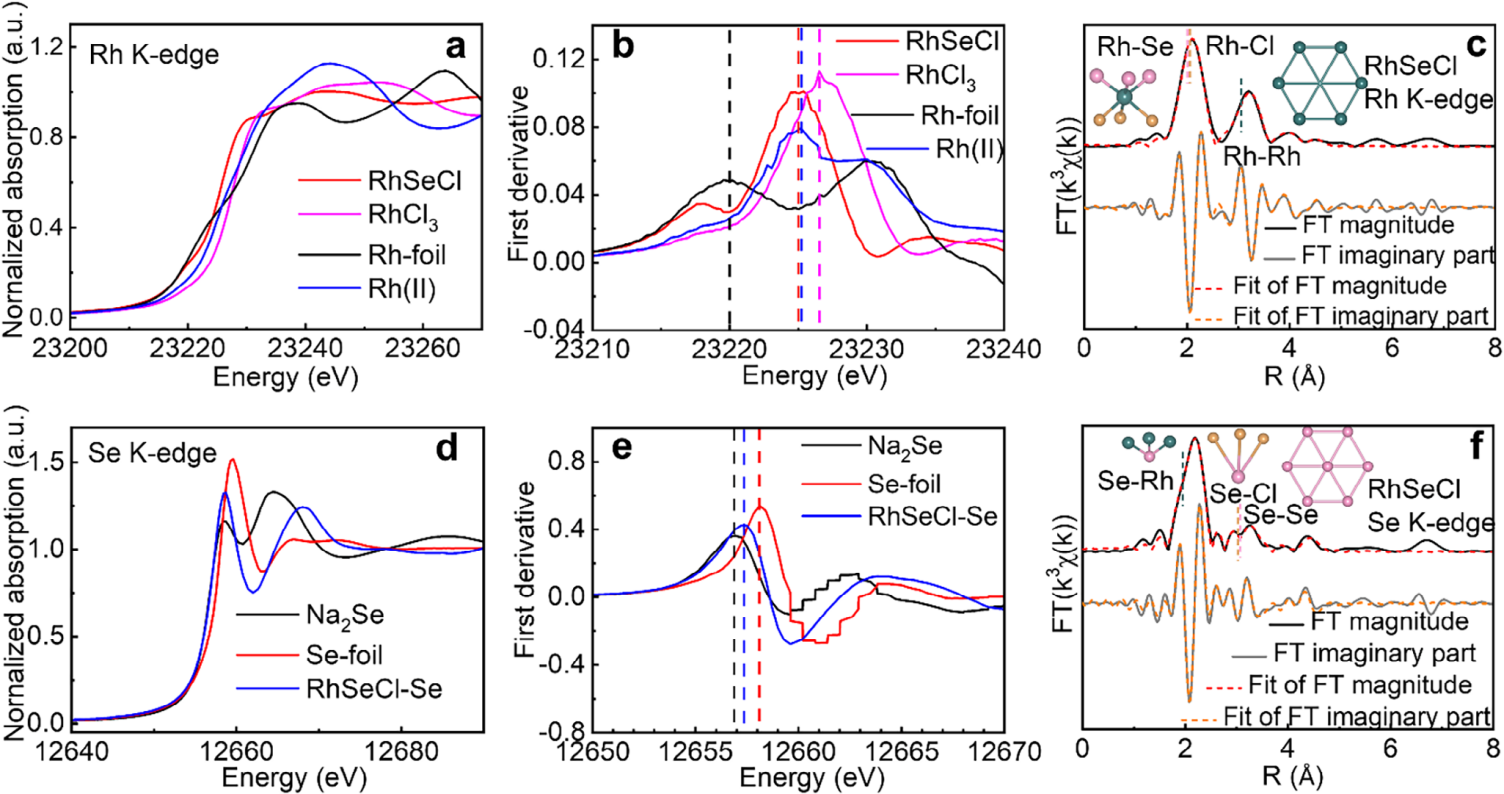
a) Rh and d) Se K‐edge X‐ray absorption near edge structure (XANES) spectra of RhSeCl and reference materials. Rh foil, rhodium(II) acetate dimer, and RhCl₃ serve as references to assess the valence state of Rh for zero, +2, and +3 oxidation states, respectively. Reference materials for Se are Se foil and Na₂Se. b) and e) are the first derivative curves of XANES spectra from a) and d). c) and f) are Fourier transformed k^3^‐weighted Rh and Se K‐edge EXAFS experimental data and the corresponding fitting results.

Understanding the oxidation states and chemical bonding in Janus compounds is crucial for elucidating their phase stability and for gaining insight into their chemical and physical properties. For Janus metal chalcohalides,^[^ ^16^ ^]^ the charge form of RhSeCl might be taken as [Rh]^3^⁺[SeCl]^3^^−^. K‐edge X‐ray Absorption Spectroscopy (XAS) measurements are performed^[^ ^30^, ^31^, ^32^ ^]^ to elucidate the chemical valence and coordination environment of Rh and Se in RhSeCl. The XANES spectrum (**Figure** **3**a,b) shows that the absorption edge of Rh in RhSeCl is between those of Rh foil and RhCl_3_, aligning closely with that of rhodium (II) acetate, indicating that Rh adopts a +2 oxidation state. Extended X‐ray absorption fine structure (EXAFS) fitting results (Figure 3c; Figure S5, and Table S7, Supporting Information) further clarify the coordination environment of Rh. The prominent peak at ≈2.0 Å corresponds to an octahedral Rh─Se/Cl coordination, with Rh─Se and Rh─Cl bond lengths of 2.41 and 2.53 Å, respectively. A peak at ≈3.2 Å is associated with hexagonal Rh─Rh coordination at ≈3.5 Å (Table S7, Supporting Information). For comparison, the Rh─Rh bond length in Rh foil is 2.69 Å, double the metallic radius of Rh (1.34 Å),^[^ ^33^ ^]^ while the Rh─Cl bond length in RhCl_3_ is shorter by 0.19 Å than in RhSeCl (Figure S6 and Tables S10 and S11, Supporting Information). The Rh^2^⁺ ionic radius is estimated to be 0.2 Å larger than that of Rh^3^⁺. For Se, the Se K‐edge absorption peak in RhSeCl falls between those of Se foil and Na_2_Se (Figure 3e), indicating that Se in RhSeCl carries a −1 oxidation state. The strongest EXAFS peak (Figure 3f) at ≈2.0 Å indicates Se is coordinated to three Rh atoms, forming a triangular pyramidal arrangement. EXAFS fitting (Tables S8 and S9, Supporting Information) confirms a Se─Rh bond length of 2.41 Å, consistent with the Rh EXAFS results. Additionally, the Se─Cl and Se─Se interatomic distances are 3.46 and 3.52 Å, respectively, with coordination numbers of 3 and 6, suggesting limited interaction between Se and surrounding Se/Cl atoms. The high‐resolution XPS spectra for Rh, Se, and Cl are highly consistent with the XAS results, as shown in Figure S7a–c (Supporting Information). Rh 3d spectra for RhSeCl were fitted with two spin–orbit doublets at 308.2 eV (Rh 3d_5/2_) and 312.9 eV (Rh 3d_3/2_), which are unexpectedly close to those of rhodium acetate (3d_5/2_ at 308.9 eV and 3d_3/2_ at 313.7 eV) but significantly lower than those in RhCl_3_ (3d_5/2_ at 310 eV and 3d_3/2_ at 314.8 eV, Figure S7d–f, Supporting Information). The Cl 2p spectrum shows peaks at 198.6 and 200.2 eV corresponding to the Cl 2p_3/2_ and Cl 2p_1/2_ transitions, respectively, indicating Cl in the −1 oxidation state. Interestingly, the Se 3d spectrum displays peaks at 55.6 and 54.8 eV, attributed to Se 3d_3/2_ and Se 3d_5/2_, respectively, indicating the presence of Se^−^, similar to RhSe_2_.^[^ ^34^ ^]^ The uniform anionic valence minimizes electrostatic repulsion between Se and Cl, thereby stabilizing the Janus configuration. These results, which differ from initial expectations, highlight the complex interactions between the transition metal and the mixed anions in RhSeCl.

To gain deeper insights into the chemical bonding, we conduct a crystal orbital Hamilton population (COHP) analysis. Table S12 (Supporting Information) presents the integrated COHP (ICOHP) values up to the Fermi level for Rh─Se and Rh─Cl interactions. The ICOHP analysis reveals that the Rh─Se bond (ICOHP = −2.78) is significantly stronger than the Rh─Cl bond (ICOHP = −1.47). This preferential bonding drives Se^−^ to occupy one face of the octahedron and Cl^−^ the opposite, generating a dipole moment that further stabilizes the Janus configuration. We also calculate the ICOHP values for Rh/Bi─Se/Te and Rh/Bi─X interactions in RhTeCl, BiSeCl, and BiTeI for comparison. The Rh─Te/Bi─Se/Bi─Te bonds are found to be stronger than the Rh─Cl/Bi─Cl/Bi─I bonds. Notably, the Rh─Se and Rh─Cl bonds exhibit the highest strength among the Rh/Bi─Se/Te and Rh/Bi─X interactions, indicating the critical role in stabilizing the structure.

### 2.3 Nonlinear Optical Feature of RhSeCl

**Figure 4:**
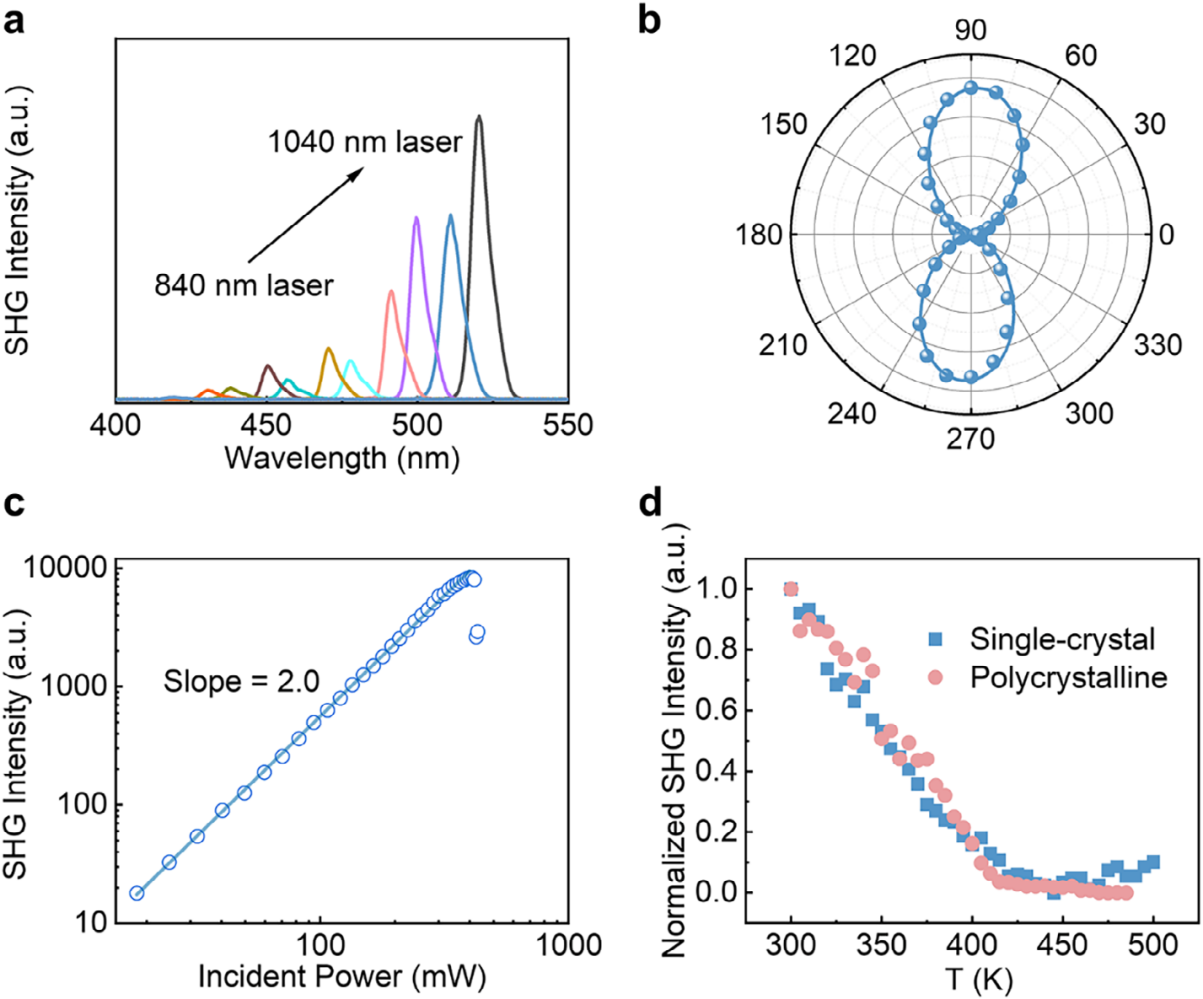
a) Wavelength‐dependent SHG spectra of RhSeCl pumped from an 840–1040 nm laser. b) SHG intensity of RhSeCl crystal at various linearly polarized angles of 1020 nm laser. The blue line fits the nonlinear data points very well. c) Logarithmic plot demonstrates the quadratic dependence of SHG intensity on incident power at 1000 nm laser, which decreases when the incident power exceeds 410 mW. d) Temperature‐dependent normalized SHG intensity of RhSeCl single crystal and polycrystalline at 1030 nm.

Second harmonic generation (SHG), a nonlinear optical (NLO) phenomenon, occurs only in materials without inversion symmetry. The observed out‐of‐plane asymmetry in RhSeCl suggests the presence of second‐order NLO responses. SHG measurements on its single crystals were performed by a home‐built femtosecond laser installation in the reflection geometry, with both incidence and detection angles of 45°.^[^ ^35^, ^36^ ^]^ The (002) plane, characterized as a large and smooth exposed surface in the morphology calculation, is chosen as the NLO test surface (Figure S8, Supporting Information). Excitation with a broadband pump laser (840–1040 nm), acting on the smooth exposed surface, generates clear SHG signals at half of the corresponding excitation wavelengths, spanning from 420 to 520 nm (**Figure** **4**a). Notably, a pronounced SHG signal occurred at 1040 nm, demonstrating the robust second‐order NLO properties of bulk RhSeCl. Furthermore, the bright signals among 1040–980 nm suggest a wide working waveband for RhSeCl. The quadratic relationship between SHG intensity and input laser power at a 1000 nm pump wavelength further confirms the two‐photon nature of the process (Figure 4c). Such a quadratic dependence maintains up to ≈410 mW, indicating a high laser‐induced damage threshold (LDT) exceeding that of the Janus MoSSe.^[^ ^37^ ^]^ Additionally, the azimuthal dependence of the SHG response in RhSeCl crystals has also been investigated by measuring SHG intensity at various linearly polarized angles. The results reveal a distinct asymmetry in SHG intensity (Figure 4b), following a dipolar profile that is well‐fitted to a cos^4^θ function.^[^ ^38^ ^]^ Intensity maxima occur at polarized angles of ≈90° and 270°, with minima at ≈0° and 180°. The polarization ratio, *ρ* = (*I_max_−I_min_*)/(*I_max_* + *I_min_*), is calculated to be ≈92%, indicating the high anisotropy under linearly polarized excitation and good crystalline character. Figure 4d presents temperature‐dependent NLO measurements conducted on both single‐crystal and polycrystalline RhSeCl from 300 to 500 K, revealing a gradual decrease in SHG intensity with increasing temperature. A similar trend has been observed in Janus TMDs, attributed to the weakening of van der Waals interactions at elevated temperatures.^[^ ^39^ ^]^

**Figure 5:**
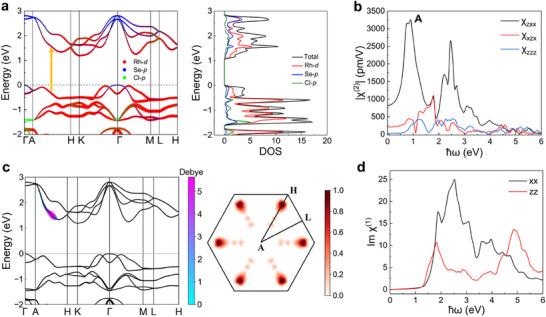
a) Projected electron band structure and density of states of bulk RhSeCl. b) Second‐order nonlinear susceptibility |*χ* ^(2)^| as a function of incident photon energy. c) Projection of the transition matrix element for peak A onto the band structure (left) and weight distribution δ_*E*_ describing the band nesting effect at various k‐points on the *k_z_* = 0.5 plane in reciprocal space (right). d) Optical absorption spectra for incident photons with varying polarization directions.

To investigate the observed SHG in RhSeCl, first‐principles calculations were conducted, revealing an indirect bandgap of 1.19 eV in the calculated electronic structure, which aligns with the measured optical bandgap value of 1.3 eV (Figure S9, Supporting Information), with the conduction band minimum (CBM) at the K point and the valence band maximum (VBM) at the Γ point (**Figure** **5**a). The SHG polarization is described by the following relation:^[^ ^40^ ^]^

(1) $$\begin{array}{c} P^{a}\;\left(2\omega \right)=\varepsilon_{0}\;\chi_{abc}^{\left(2\right)}E^{b}\left(\omega \right)E^{c}\left(\omega \right) \end{array}$$

where ε_0_ denotes the dielectric constant in vacuum, χ^(2)^ represents the second‐order nonlinear susceptibility, the *a*, *b*, and *c* refer to the components along the Cartesian coordinates, and *E*(ω) signifies the electric field component of the incident photon at frequency ω. Bulk RhSeCl adopts a hexagonal crystal structure, belonging to point group 6 mm and the space group *P*6₃*mc*. Based on structural symmetry and intrinsic commutation symmetry, $\chi_{abc}^{\left(2\right)}$ of bulk RhSeCl has three non‐zero independent tensor components, which are: χ_*xzx*_ = χ_*xxz*_  = χ_*yyz*_  = χ_*yzy*_ , χ_*zxx*_ = χ_*zyy*_ and χ_*zzz*_. Notably, the χ_*zxx*_ component exhibits a substantially higher magnitude compared to the other two non‐zero independent components. Specifically, peak A is located at ≈0.9 eV, with an amplitude exceeding 3000 pm V^−1^, indicating a strong nonlinear response. For the same component ($\chi_{zxx}^{\left(2\right)}$), the overall value of RhSeCl is much larger than that of monolayer MoSSe.^[^ ^41^ ^]^

As a typical example, we demonstrate the origin of peak A in the χ_*zxx*_ component. To elucidate the SHG mechanism, we calculate the transition dipole moment, a key factor influencing SHG, as shown in the left panel of Figure 5c. The transition for peak A predominantly occurs along the A‐H high‐symmetry points, indicated by the yellow arrow in Figure 5a. This transition involves a two‐photon resonance primarily associated with the Rh *d*‐orbitals, linking the degenerate valence bands to the conduction bands. Additionally, we observe that the valence and conduction bands are nearly parallel at these transition points, leading to a significant band nesting effect.^[^ ^42^, ^43^ ^]^ This band nesting strongly enhances the nonlinear optical response at this energy level. To quantify the band nesting at peak A, we define a delta‐like function as:

(2) $$\begin{array}{c} \delta_{E}\left(k\right)=1-\frac{\left|E_{CB}\left(k\right)-E_{VB}\left(k\right)-2\hbar \omega_{peak\;A}\right|}{0.04} \end{array}$$

where *E*(*k*) represents the energy of different k points, 2ℏω_*peak* *A*_ represents the double‐frequency energy of the incident photon corresponding to peak A, and the constant 0.04 in the denominator represents the energy broadening range. The right panel of Figure 5c shows a substantial band nesting effect along the A‐H path in the Brillouin zone, leading to the formation of numerous electron‐hole pairs at the same energy and significantly increasing the first‐order absorption intensity (Figure 5d). This finding underscores the exceptional nonlinear optical properties of Janus RhSeCl, particularly its pronounced SHG response, offering promising potential for the development of efficient optoelectronic devices. To further investigate the influence of interlayer interactions on the SHG response of bulk RhSeCl, we artificially modified their strength using a Hamiltonian constructed with the WANNIER90 code.^[^ ^44^ ^]^ This approach enables us to analyze the variation of peak A under different interlayer interaction strengths (Figure S10, Supporting Information).^[^ ^45^ ^]^ Notably, as interlayer coupling weakens—likely due to temperature‐induced interlayer expansion—the SHG intensity decreases rapidly, in agreement with experimental observations. These findings support the critical role of interlayer interactions in Janus materials and provide valuable insights for tuning their SHG response.

### 2.4 Optoelectronic Device of RhSeCl

**Figure 6:**
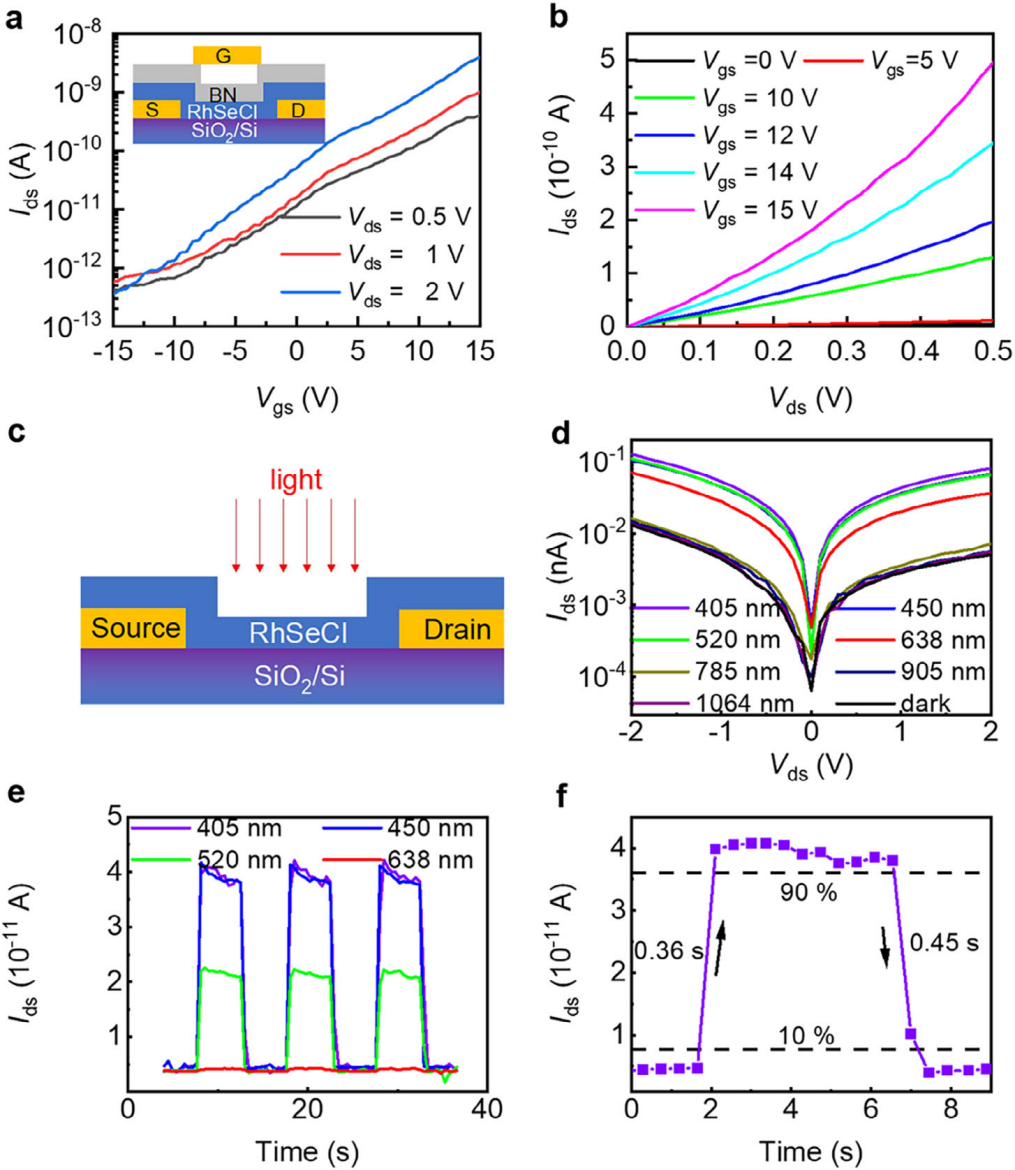
a) Transfer characteristic (*I*_ds_ – *V*_gs_) of the RhSeCl FET with *V*_gs_ between −15 and 15 V at a bias voltage range from 0.5 to 2 V in log plot. The inset shows the diagram/figure of the FET. b) *I*_ds_ – *V*_ds_ characteristics for various *V*_gs_ values. c) Schematic diagram of the photodetector. d) *I*_ds_ – *V*_ds_ curves with various laser wavelengths at a power density of 30 mW cm^−2^. e) Time‐dependent photocurrent at 1 V bias with power density of 30 mW cm^−2^. f) Rise and decay time at a light wavelength of 405 nm.

The distinctive structural features of Janus compounds frequently offer significant potential for improved optoelectronic properties.^[^ ^46^ ^]^ To explore this in RhSeCl, we have fabricated two kinds of devices to evaluate the optoelectronic performance. A RhSeCl field‐effect transistor (FET) was constructed on a SiO_2_/Si substrate using BN as top gate dielectric (inset, **Figure** **6**a). The transfer characteristics (Figure 6a) show that at a bias voltage of 2 V, the device exhibits an on‐state current *I*_on_ of 4 nA and an off‐state current *I*_off_ below 0.36 pA, with a gate leakage current of less than 0.74 pA. The calculated current on/off ratio (*I*_on_/*I*_off_) is ≈1.1 × 10^4^ over a ±15 V range, indicating comparable device performance. Furthermore, the drain‐source current as a function of voltage (*I*_ds_‐*V*_ds_) at different gate voltages (*V*_gs_) (Figure 6b) reveals effective current modulation. For RhSeCl FET, *I*_on_ is ≈0.5 nA at *V*_gs_ = 15 V and *V*_ds_ = 0.5 V. But the nonlinear *I*_ds_‐*V*_ds_ relationship at varying *V*_gs_, suggests the presence of a substantial Schottky barrier at RhSeCl and the Ti/Au metal electrode interface, indicating n‐type charge transport characteristics. The optoelectronic properties of RhSeCl are further evaluated using the device shown in Figure 6c. The *I*_ds –_ *V*_ds_ curves under dark conditions and different illumination wavelengths (Figure 6d) demonstrate a clear photoresponse at 405, 450, 520, and 638 nm, while weaker responses are observed at 785, 905, and 1064 nm. The presence of Schottky contacts between the device and Au electrodes is evident from the significant variation in *I*_ds_ over the −2–2 V range. The time response (Figure 6e) confirms the stability and repeatability of the photoresponse, as *I*_ds_ repeatedly switches on and off in sync with laser modulation. At a power density of 30 mW cm^−2^ and a 1 V bias, the switching ratios for 405, 450, 520, and 638 nm are 9.4, 8.7, 5.7, and 1.2, respectively, indicating its promising modulation capability. The rise and decay times, defined as the duration for the photocurrent to transition between 10% and 90% of its stabilized value, are measured at 0.36 and 0.45 s, respectively (Figure 6f). This indicates efficient charge carrier separation, where photogenerated electron‐hole pairs are effectively driven apart under the applied electric field.

**Figure 7:**
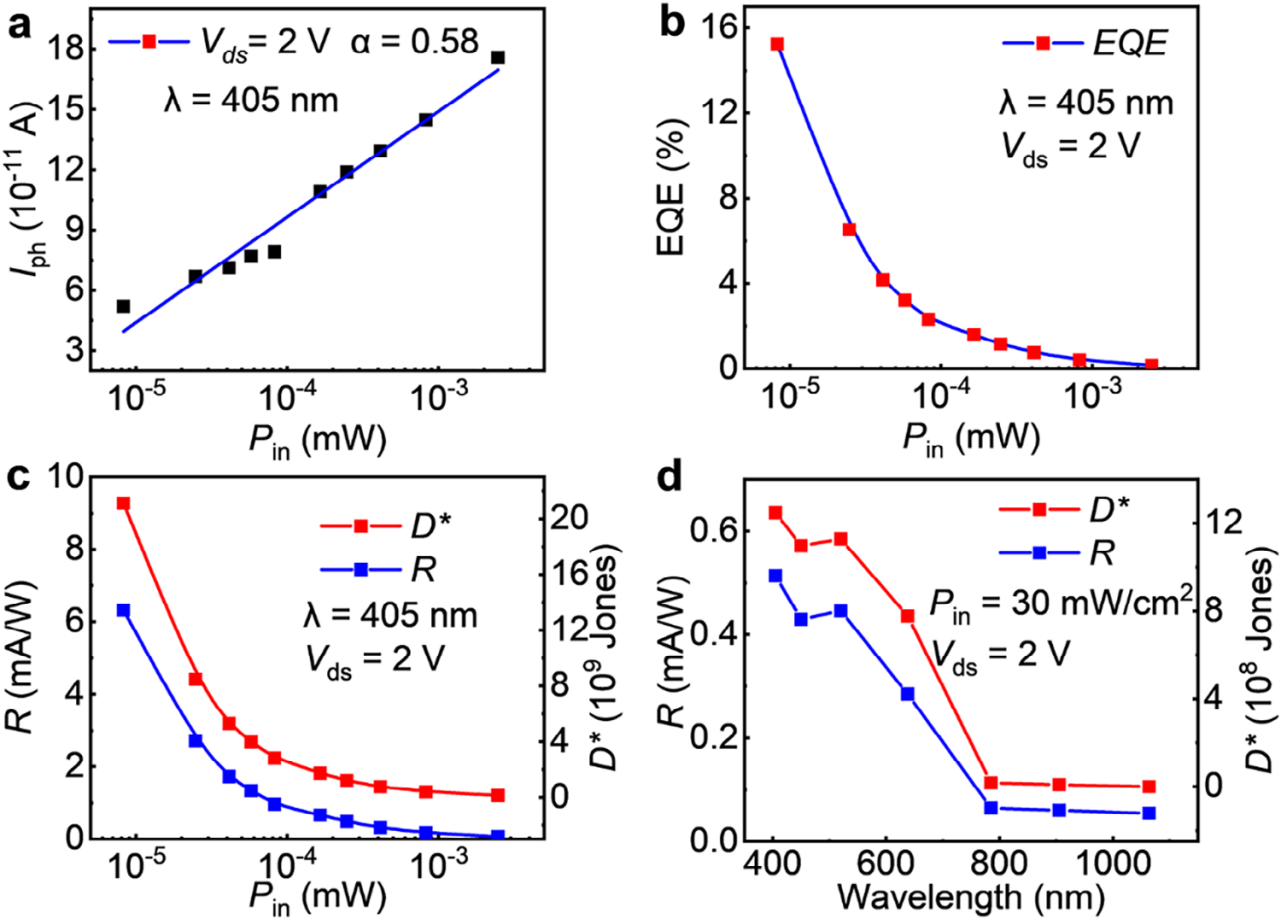
a) The relationship between *I*_ph_ and *P*_in_. b) The variation of EQE with incident light power. c) The value of *R* and D^*^ increases with the increase in incident light power. d) The variation of *R* and D^*^ with incident light wavelength.

**Figure** **7**a illustrates the relationship between the photocurrent (*I*_ph_) and the incident light power (*P*_in_) at 2 V biases. We fit the data points related to the power density at 405 nm light wavelength by the equation *I*_ph_ ∝ *P^α^*, where α is the power‐law exponent. The calculated value of α is 0.58, indicating the presence of trap states and defects at the interface between the device and the Au electrode, leading to a sublinear dependence of photogenerated electron–hole pairs on optical power. The external quantum efficiency (EQE) as a function of incident power at 405 nm (*V*_ds_ = 2 V) is shown in Figure 7b. The EQE is calculated as EQE = *hc*/*λe* × *R* × 100%, where *h* is Planck's constant, *e* is the elementary charge, *c* is the speed of light, *λ* is the wavelength. Under 405 nm laser illumination at 1 mW cm^−2^ and *V*_ds_ = 2 V, the responsivity reaches 15.25%. The responsivity (*R*) and specific detectivity (D*) are key parameters for evaluating photodetectors. *R* is determined by *R* = *I*_ph_/*P*_in_, where D^*^ can be calculated by D^*^ = *R*/(*R'A*/4*k*_B_ *T*)^1/2^, where *R’* is the bias resistance, *A* is the effective area of the device, *k*_B_ is the Boltzmann constant, and *T* is the temperature.^[^ ^47^ ^]^ As shown in Figure 7c,d and D* reaches 2.1 × 10^10^ cm Hz^1/2^ W^−1^ under 405 nm illumination at 1 mW/cm^2^ and *V*_ds_ = 2 V. Notably, although the number of photogenerated electron–hole pairs increases with the optical power, the complexation of additional electron‐hole pairs at the defects reduces the proportion of effective photogenerated electron‐hole pairs participating in conduction, resulting in decreased *R* and EQE at higher laser power.^[^ ^48^ ^]^ Moreover, the device demonstrates moderate responsivity and specific detectivity across the 405–650 nm wavelength range, demonstrating strong potential for optoelectronic applications. Its performance is expected to be further enhanced through the construction of heterostructures.^[^ ^49^, ^50^ ^]^

## 3 Conclusion

In summary, through the synthetic optimization, we have successfully synthesized the large Janus transition metal chalcohalide RhSeCl crystals using chemical vapor transport (CVT). X‐ray diffraction and HAADF‐STEM confirmed the ordered arrangement of anions, with Se and Cl positioned in a facial configuration. Comparative structure analysis suggests that size effects play a crucial role in stabilizing these structures. XANES and XPS measurements further validated the presence of Se in the unusual ‐1 oxidation state, which is attributed to the complex interactions between the mixed anions and the central metal cations. Interestingly, the second‐harmonic generation (SHG) signals were clearly observed in experiments for RhSeCl and accompanied by a noticeable decrease in intensity as the temperature increased. Theoretical calculations suggested a strong SHG response in bulk RhSeCl, with a large second‐order nonlinear susceptibility of ≈3000 pm V^−1^, which is attributed to the band nesting effect, while the temperature‐induced reduction of SHG intensity is related to the weakening of interlayer coupling strength. Furthermore, the RhSeCl exhibits superior optoelectronic performance at visual light region. Our findings offer deeper insights into 2D Janus materials and broaden their potential applications in nonlinear optics, optoelectronics, catalysis, and thermoelectrics.

## 4 Experimental Section

### Synthesis

Chemical vapor transport (CVT) and conventional solid‐state reaction were used for the synthesis of RhSeCl single crystals and polycrystalline samples, respectively. The reactants Rh (Maklin, powder, 99.95%), Se (Alfa, powder, 99.999%), and RhCl_3_ (Aladdin, powder, 99%), which acts as a chlorine source, were finely mixed in a molar ratio of 2 (0.6 mmol):3 (0.9 mmol):1.1 (0.33 mmol). For CVT method, the mixture was sealed in the fused vacuum silica ampoules (*l* = 11 cm, Ø_inner_ = 13 mm) and then put into a two‐zone furnace with a temperature gradient of Δ*T* = 70 K (*T*1 = 930 °C, *T*2 = 1000 °C) at a ramping rate of 2 K·min^−1^, followed by a longtime dwelling for 30 days and cooled to 400 °C for 48 h. Isopropanol was transferred to an ultrasonic bath (for 5 min) to remove the shiny crystals from the ampoules. For the polycrystalline preparation, the sealed precursors were sintered at 950 °C for 5 days and then naturally cooled to room temperature. A trace amount of Rh metal was detected in the PXRD (Figure S2, Supporting Information), which was primarily attributed to the exceptionally high melting point of rhodium or the high volatility of the RhCl₃ precursor, leading to potential Rh‐rich stoichiometric deviations during high‐temperature reactions.

### Single‐Crystal and Powder X‐Ray Diffraction

Variable temperature single‐crystal structures for RhSeCl were systematically collected within the temperature regime from 30 to 500 K. The data of 30 K were collected on a Bruker D8 Venture diffractometer (Mo‐Kα radiation λ = 0.71073 Å). The data collection and reduction were processed by the APEX 3 software package,^[^ ^51^ ^]^ v2018.1‐0. For the X‐ray diffraction experiments from 100 to 500 K, a Rikagu XtaLAB Synergy R HyPix diffractometer (Mo‐Kα radiation λ = 0.71073 Å) was used in conjunction with CrysAlis^Pro^ software,^[^ ^52^ ^]^ version 43.129a. These structures were initially solved by intrinsic phasing methods and further refined using SHELXL 2017/1^[^ ^53^ ^]^ by full‐matrix least‐squares against *F^2^*. Anisotropic thermal parameters were applied to all the atoms. Powder X‐ray diffraction patterns were recorded on the PANalytical Empyrean diffractometer with Cu K*α* radiation (λ = 1.5418Å), in a range of 5°≤ 2θ≤ 120° and a scan speed of 1° min^−1^. Rietveld refinement was performed using Fullprof Suit Toolbar 64‐bit.

### Morphological Characterization

A scanning electron microscope (FESEM, JSM‐7900F, JEOL Co., Ltd., Tokyo, Japan) equipped with Energy Dispersive Spectroscopy (EDS), was used to observe the morphologies and the elements distribution map of the samples with an acceleration voltage of 15 kV. STEM characterization was performed using a JEOL JEM‐ARM300F2 spherical‐aberration‐corrected scanning transmission electron microscope, equipped with an EDS detector and operated at an accelerating voltage of 300 kV. The probe convergence angle was adjusted to 25 mrad, while the HAADF imaging collection angle ranged from 65 to 200 mrad.

### Chemical States and Bonding

The surface electronic states of RhSeCl material were performed on the Thermo Scientific ESCALab 250Xi X‐ray photoelectron spectroscopy (XPS) with 150 W monochromatic Al Kα (*hν* = 1486.6 eV) radiation. Typically, the hydrocarbon C1s line at 284.8 eV from adventitious carbon was used for energy referencing. X‐ray absorption spectroscopy (XAS) data were collected in the transmission mode at Beamline 11B of the Shanghai Synchrotron Radiation Facility (SSRF). The current of the storage ring was ≈180 mA, using a Si (311) double crystal monochromator to generate monochromatic X‐rays. The energy calibrations of the monochromator were verified by the K‐edges of Rh foil (23220 eV) and Se foil (12658 eV). All XAS data were analyzed by the IFEFFIT software suite and the WinXAS program.^[^ ^54^, ^55^ ^]^

### Nonlinear Optical Properties

The nonlinear optical (NLO) measurements of the RhSeCl crystals were performed with a home‐built scanning microscope with a femtosecond laser pump (Mai Tai HP,<100 fs, 80 MHz, 690–1040 nm) in reflection geometry, with the incidence and detection angles both at 45°. The linearly polarized pumps were tuned by a half‐wave (λ/2) plate. Signals were collected from the front surfaces, with both the incident light and the reflected SHG signals being *p*‐polarized. The in situ SHG spectra in a back reflection configuration were measured by a SHG detection system (gora‐Lite, ideaoptics, China). A femtosecond (fs) laser with a wavelength of 1030 nm, a repetition rate of 25 MHz, and a pulse width of 180 fs was employed as the exciting light source. In the experiment, the SHG intensity was characterized by the intensity of the outgoing light (I).

### Device Fabrication and Characterization

RhSeCl flakes were cleaved from bulk crystals onto polydimethylsiloxane (PDMS) by the mechanical exfoliation method. Then RhSeCl flake was transferred to a 285 nm SiO_2_/Si substrate with pre‐patterned electrodes, respectively. The Ti/Au (10/40 nm) electrodes were fabricated by electron beam lithography, and metals were deposited using the thermally evaporating method. BN was transferred to RhSeCl using PDMS as the top gate. BN was then connected to the electrodes by graphene. The electronic and optoelectronic properties were measured by using a semiconductor parameter analyzer (4200‐SCS, Keithley).

### DFT Calculations

Structural optimization and electronic properties were carried out using the Vienna Ab initio Simulation Package (VASP)^[^ ^56^ ^]^ employing the Projected Augmented Wave (PAW)^[^ ^57^ ^]^ method within the framework of Density Functional Theory (DFT).^[^ ^58^, ^59^ ^]^ The exchange‐correlation interaction of electrons was treated using the Perdew–Burke–Ernzerhof (PBE) functional within the generalized gradient approximation (GGA).^[^ ^60^ ^]^ The cutoff energy was set to 350 eV, with convergence criteria for total energy and stress fixed at 1 × 10^−6^ eV and 0.02 eV Å^−1^, respectively. A gamma‐centered k‐point grid density of 15 × 15 × 5 was used for Brillouin zone sampling. The above parameter settings could ensure the reliable convergence of the calculation results. The DFT‐D3^[^ ^61^ ^]^ method of Grimme was used to correct the interlayer van der Waals interaction. The crystal orbital Hamilton populations (COHP) are employed as implemented in the LOBSTER package.

For the calculation of nonlinear optical properties, the in‐house developed package, NOPSS was employed based on the aforementioned computational framework. This package has undergone rigorous testing to ensure the accuracy of the computational results.^[^ ^62^ ^]^ Due to the higher k‐point density required for optical properties, a gamma‐centered 24 × 24 × 10 k‐point mesh was utilized and 80 bands were calculated to guarantee the convergence of the results (Figure S11, Supporting Information). Additionally, an incident photon energy range of 0–6 eV was selected, with 200 sampling points to ensure sufficient smoothness of the spectral lines.

The $\chi_{abc}^{\left(2\right)}$ can be expressed as:^[^ ^40^, ^63^ ^]^

(3) $$\begin{array}{c} \chi_{abc}^{\left(2\right)}\;\left(-2\omega ,\omega ,\omega \right)=\chi_{inter}^{abc}\;\left(-2\omega ,\omega ,\omega \right)+\chi_{intra}^{abc}\left(-2\omega ,\omega ,\omega \right) \end{array}$$

Here, the first term on the right‐hand side represents the second‐order nonlinear susceptibility tensor contribution purely from interband transitions, while the second term arises from mixed contributions of both interband and intraband transitions. The terms $\chi_{inter}^{abc}\left(-2\omega ,\omega ,\omega \right)$ and $\chi_{intra}^{abc}\left(-2\omega ,\omega ,\omega \right)$ can be represented as follows:

(4) $$\chi_{\mathrm{inter}}^{\mathrm{abc}}\;\left(-2\omega ,\omega ,\omega \right)=\begin{array}{c} \frac{e^{3}}{\hbar^{2}\Omega }\sum_{\mathrm{nml},k}\frac{r_{\mathrm{nm}}^{a}\left\{r_{\mathrm{ml}}^{b}r_{\ln}^{c}\right\}}{\left(\omega_{\ln}-\omega_{\mathrm{ml}}\right)}\times \left[\frac{2f_{\mathrm{nm}}}{\omega_{\mathrm{mn}}-2\omega }+\frac{f_{\ln}}{\omega_{\ln}-\omega }+\frac{f_{\mathrm{ml}}}{\omega_{\mathrm{ml}}-\omega }\right] \end{array}$$

(5) $$\begin{array}{ccc} & & \chi_{intra}^{abc}\;\left(-2\omega ,\omega ,\omega \right)=\frac{i}{2}\frac{e^{3}}{\hbar^{2}\Omega }\sum_{nm,k}f_{nm}\left[\frac{2}{\omega_{mn}\left(\omega_{mn}-2\omega \right)}r_{nm}^{a}\left(r_{mn;c}^{b}+r_{mn;b}^{c}\right)\right.\\ & & +\left.\frac{1}{\omega_{mn}\left(\omega_{mn}-\omega \right)}\left(r_{nm;c}^{a}r_{mn}^{b}+r_{nm;b}^{a}r_{mn}^{c}\right)+\frac{1}{\omega_{mn}^{2}}\left(\frac{1}{\omega_{mn}-\omega }-\frac{4}{\omega_{mn}-2\omega }\right)\right.\\ & & \times \left.r_{nm}^{a}\left(r_{mn}^{b}\Delta_{mn}^{c}+r_{mn}^{c}\Delta_{mn}^{b}\right)-\frac{1}{2\omega_{mn}\left(\omega_{mn}-\omega \right)}\left(r_{nm;a}^{b}r_{mn}^{c}+r_{nm;a}^{c}r_{mn}^{b}\right)\right] \end{array}$$

Here, the subscripts *n*, *m* and *l* represent the band indices, and *a*, *b* and *c* denote the Cartesian coordinate indices. The term $r_{nm}^{a}$ represents transition matrix element. *f* is the Fermi distribution function, where *f_nm_* = *f_n_*  − *f_m_*. The symbol *ω* refers to the frequency, with ω_*nm*_ = ω_*n*_  − ω_*m*_. Ω represents the volume of the unit cell. The term $r_{nm;c}^{b}$ denotes the generalized derivative of the dipole matrix element, using its sum role^[^ ^64^ ^]^ given by: $r_{nm;c}^{b}=\frac{r_{nm}^{c}\Delta_{mn}^{b}+r_{nm}^{b}\Delta_{mn}^{c}}{\omega_{nm}}+\frac{i}{\omega_{nm}}\times \sum_{l}\omega_{lm}r_{nl}^{c}r_{lm}^{b}-\omega_{nl}r_{nl}^{b}r_{lm}^{c}\cdot \Delta_{mn}^{c}$ represents the difference in the electron group velocities, defined as $\Delta_{mn}^{c}=v_{mm}^{c}\;-v_{nn}^{c}$.

## Conflict of Interest

The authors declare no conflict of interest.

## Author Contributions

K.L., X.S., and P.C. contributed equally to this work. H.G. conceived the concept. H.G., B.H., and X.Z. designed the project and supervised the work. K.L. conducted synthesis experiments. K.L. and D.J. conducted the single crystal analysis. K.L., P.L., and A.N. conducted the morphological characterization. K.L., S.Z., S.Z., Y.C., T.G., and J.L. conducted the chemical states characterization. P.C. and X.W. conducted the nonlinear optical properties experiments. Z.L. conducted the fabrication and characterization of optoelectronic devices. X.S., X.Y., and L.Y. conducted the theoretical calculations. K.L., X.S., P.C., Z.L., S.Z., S.X., J.X., X.Z., B.H., and H.G. wrote the manuscript with inputs from all the coauthors.

## Supporting information



Supporting Information

## Data Availability

The data that support the findings of this study are available from the corresponding author upon reasonable request.
