# Supplementary material for: Optimized Synthesis and Characterization of Janus RhSeCl with Uniform Anionic Valences, Nonlinear Optical and Optoelectronic Properties
Source: Adv Sci (Weinh). 2025 Jun 20;12(34):e05279. doi: 10.1002/advs.202505279 (PMC12442689; doi:10.1002/advs.202505279)
Supplement: Supplementary file 1 — Supporting Information [file ADVS-12-e05279-s001.docx]

**Optimized Synthesis and Characterization of Janus RhSeCl with Uniform Anionic Valences, Nonlinear Optical and Optoelectronic Properties**

Kefeng Liu,^†[1]^ Xuelian Sun,^†[2]^ Puxin Cheng,^†[3]^ Zhiteng Li,^[4]^ Penghui Li,^[5]^ Donghan Jia,^[1]^ Shijing Zhao,^[1]^ Xin Yang,^[1]^ Xinyu Wang,^[1]^ Liangting Ye,^[2]^ Shengqing Xia,^[6]^ Shuo Zhang,^[7]^ Yu Chen,^[7]^ Tao Gan,^[7]^ Jiong Li,^[7]^ Xiao Zhang,^*[4]^ Jialiang Xu,^[3]^ Anmin Nie,^[5]^ Bing Huang^*[2],[8]^ and Huiyang Gou^*[1]^

[1] Dr. K. Liu, D. Jia, Dr. S. Zhao, Dr. X. Yang, X. Wang, Prof. Dr. H. Gou^*^
Center for High Pressure Science and Technology Advanced Research (HPSTAR)
Beijing 100094 (P. R. China)
^*^E-mail: huiyang.gou@hpstar.ac.cn; huiyang.gou@gmail.com

[2] X. Sun, L. Ye, Prof. B. Huang^*^

Beijing Computational Science Research Center

Beijing 100193 (P. R. China)
^*^E-mail: bing.huang@csrc.ac.cn

[3] P. Cheng, Prof. J. Xu
School of Materials Science and Engineering
Tianjin Key Laboratory of Metal and Molecular Materials Chemistry

Frontiers Science Center for New Organic Matter

Nankai University

Tianjin 300350 (P. R. China)

[4] Z. Li, Prof. X. Zhang^*^

State Key Laboratory of Information Photonics and Optical Communications

School of Physical Science and Technology

Beijing University of Posts and Telecommunications

Beijing 100876 (P. R. China)
^*^E-mail: zhangxiaobupt@bupt.edu.cn

[5] P. Li, Prof. A. Nie

Center for High Pressure Science

State Key Laboratory of Metastable Materials Science and Technology

Yanshan University

Qinhuangdao 066044 (P. R. China)

[6] S. Xia

State Key Laboratory of Crystal Materials

Institute of Crystal Materials

Shandong University

Shandong 250100 (P. R. China)

[7] S. Zhang, Y. Chen, T. Gan, J. Li

Shanghai Synchrotron Radiation Facility

Shanghai Advanced Research Institute

Chinese Academy of Sciences

Shanghai 201204 (P. R. China)

[8] Prof. B. Huang^*^

Department of Physics

Beijing Normal University

Beijing 100875 (P. R. China)
^*^E-mail: bing.huang@csrc.ac.cn

^†^Kefeng Liu, Xuelian Sun and Puxin Cheng contributed equally to this work

**
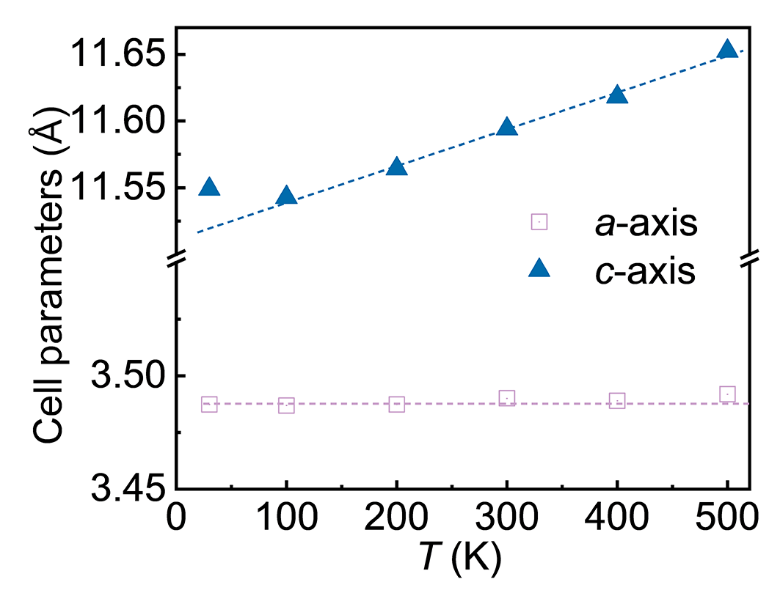
**

**Figure S1.** Temperature-dependent cell parameters in RhSeCl from 30 K to 500 K.

**
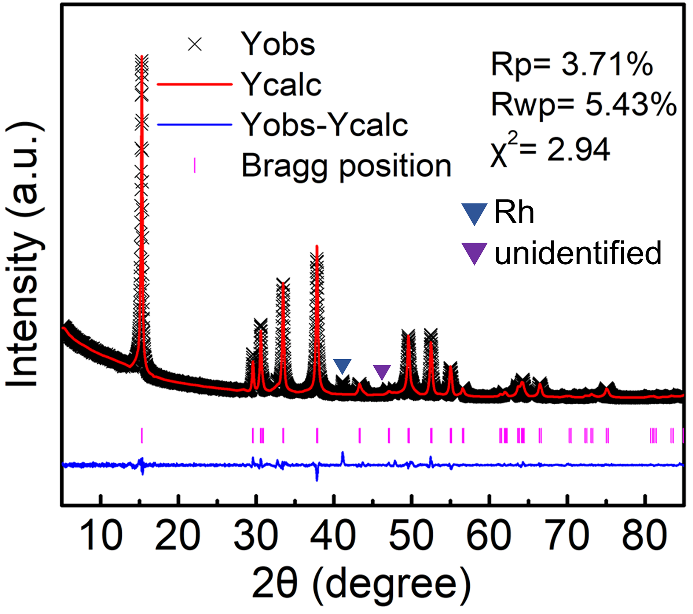
**

**Figure S2.** The Rietveld refinement of X-ray powder diffraction pattern for RhSeCl. There is a trace mount of Rh byproduct detected, in which the diffraction range from 40.7° to 41.3° was excluded when performing refinement.


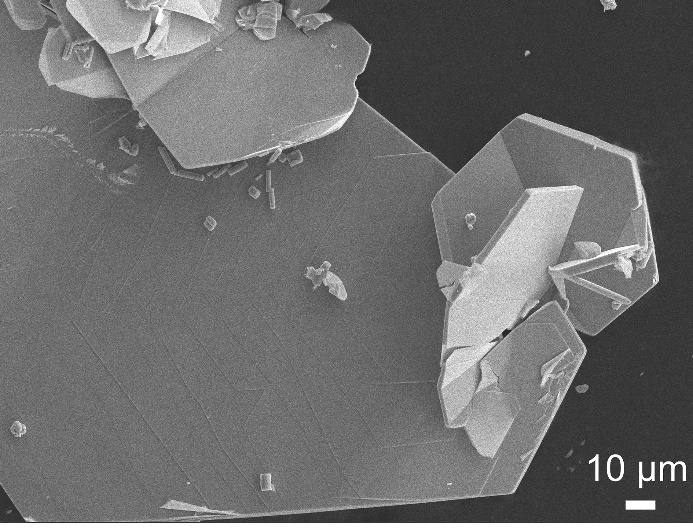


|  | Rh /At-% | Se /At-% | Cl /At-% |
| --- | --- | --- | --- |
| 1 | 34.68 | 32.42 | 32.90 |
| 2 | 34.10 | 32.47 | 33.43 |
| 3 | 33.65 | 34.07 | 32.28 |
| 4 | 36.43 | 33.15 | 30.42 |
| 5 | 34.73 | 34.88 | 30.39 |
| Average | 34.72 | 33.40 | 31.88 |

**Figure S3.** SEM image of grown single crystals and atomic distribution of component elements.

**
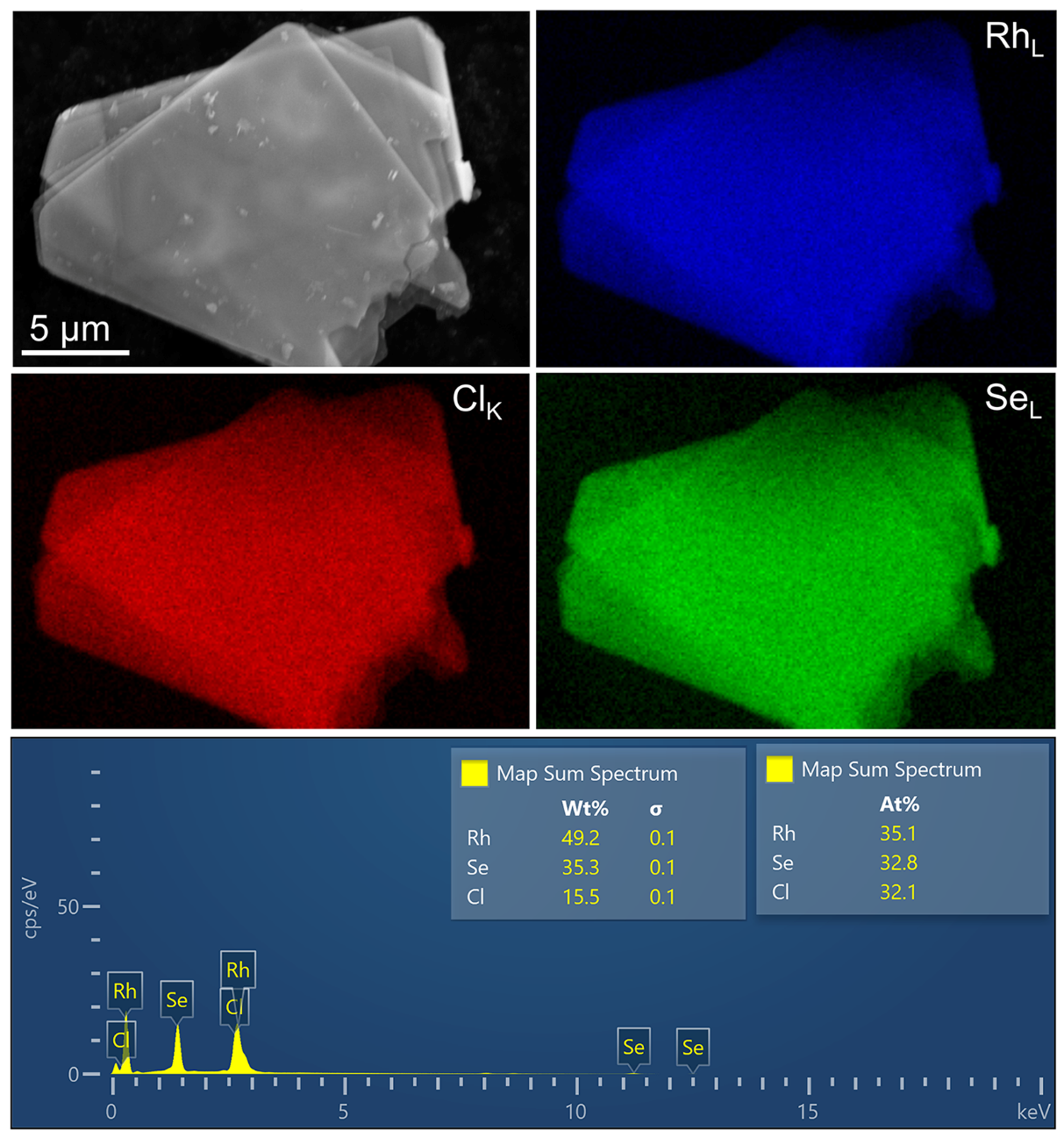
**

**Figure S4.** EDX spectroscopy mapping of the component elements of RhSeCl. These results indicate a uniform distribution of the elements.

**
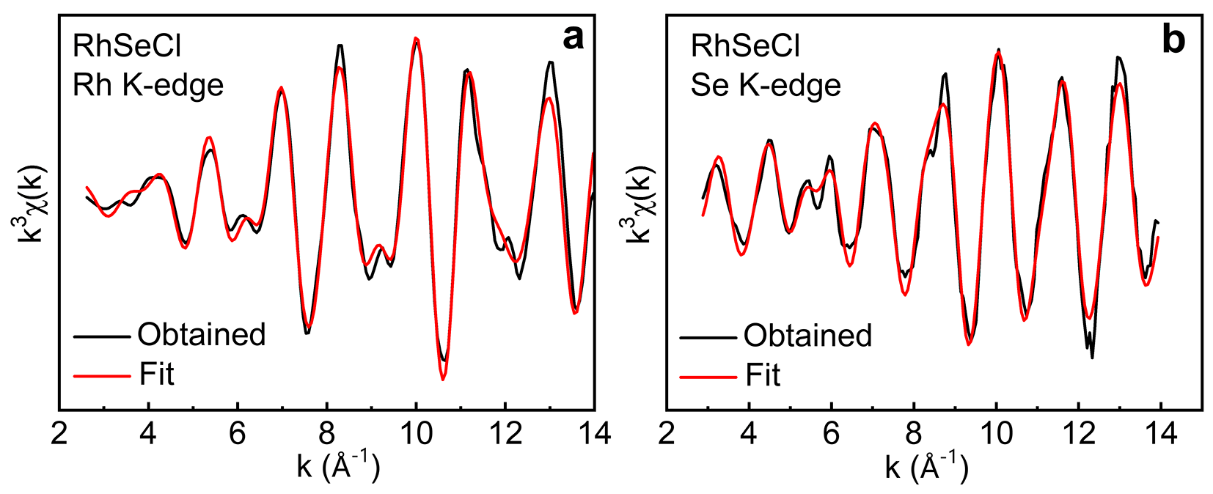
**

**Figure S5.** k^3^ weighted χ(k) spectra of Rh and Se K-edge in RhSeCl. The red lines are fitted curves.

**
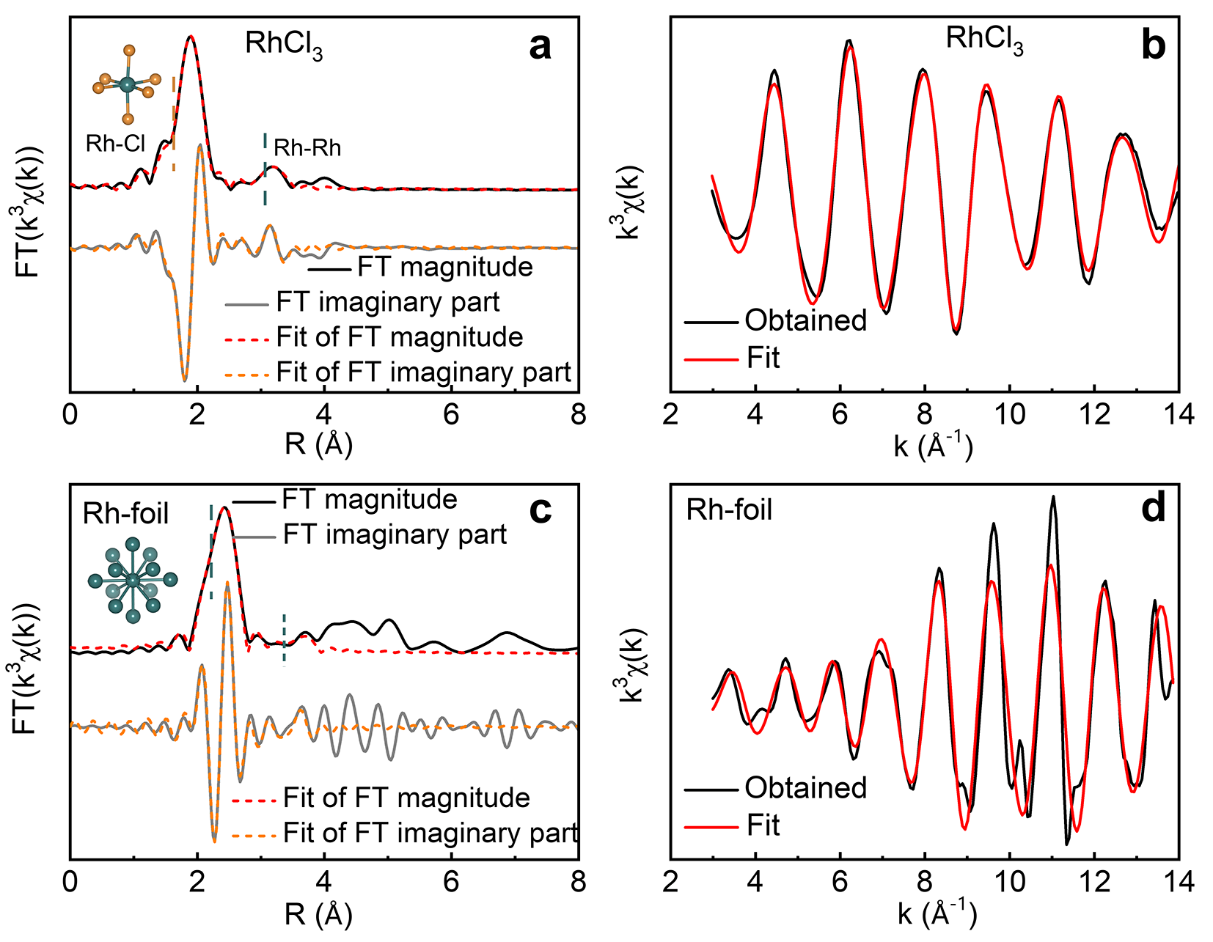
**

**Figure S6.** Fitting results of Rh K-edge EXAFS for RhCl_3_ and Rh foil. (a) and (c) are Fourier transformed k^3^-weighted Rh K-edge EXAFS experimental data and the corresponding fitting results of RhCl_3_ and Rh foil. (b) and (d) are k^3^ weighted χ(k) Rh K-edge spectra of RhCl_3_ and Rh foil, and the red lines are fitted curves.


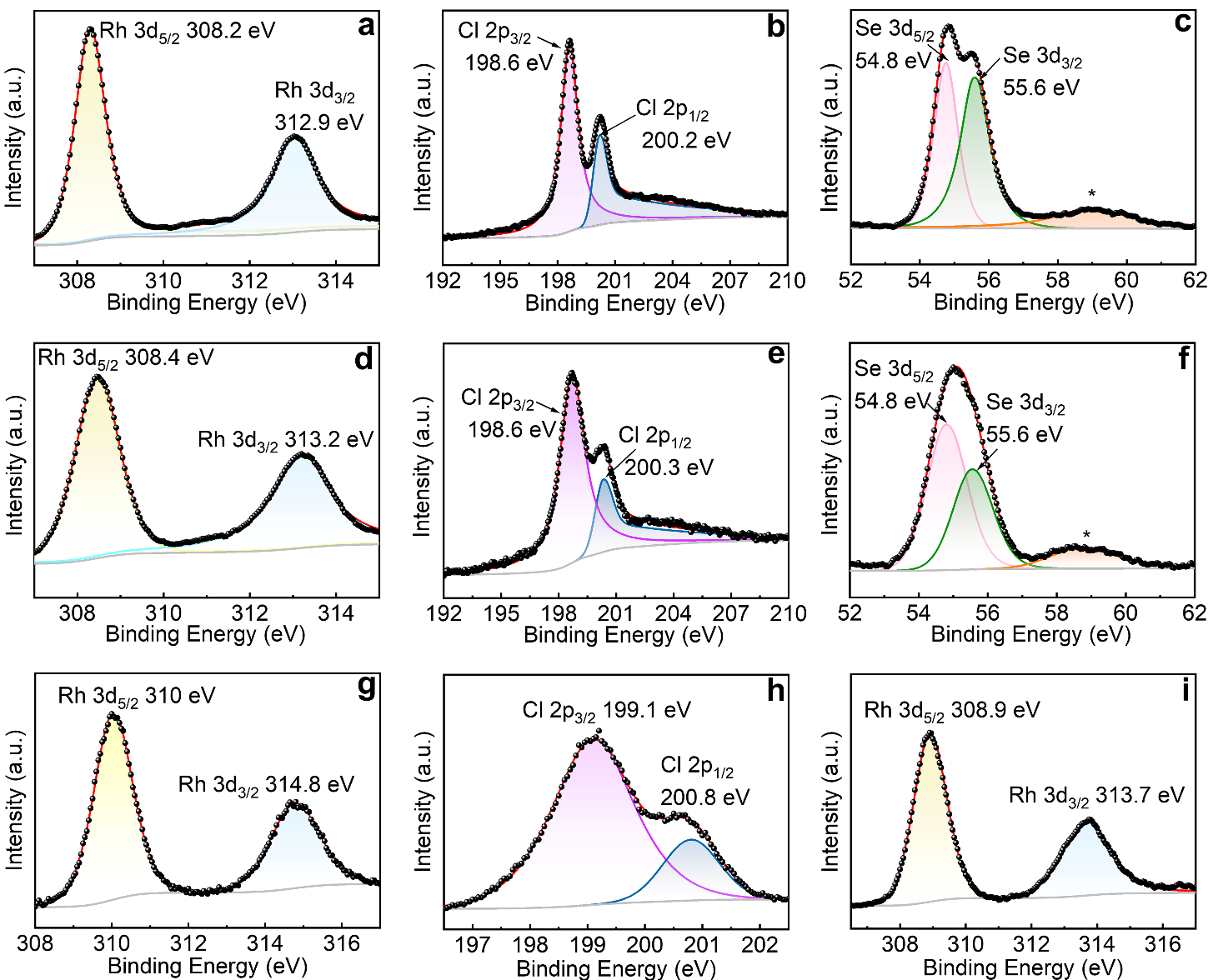


**Figure S7.** High-resolution XPS spectra. (a)-(c) Spectra for Rh 3d, Cl 2p and Se 3d in freshly synthesized RhSeCl. (d)-(f) Spectra for Rh 3d, Cl 2p and Se 3d in exposed RhSeCl for three months. After exposure, the Rh 3d_5/2_, Se 3d_5/2_ and Cl 2p_3/2_ peak positions shifted by <0.3 eV. The Cl 2p/Rh 3d intensity ratio remained effectively unchanged (0.23 ± 0.07 before vs. 0.24 ± 0.05 after exposure), indicating minimal surface halide loss. The broad peak in (c) and (f) denoted by “*” at 58.9 eV may be related to SeO_2_. (g) and (h) Spectra for Rh 3d and Cl 2p in RhCl_3_. (i) Spectra for Rh 3d in Rhodium(II) acetate.

**
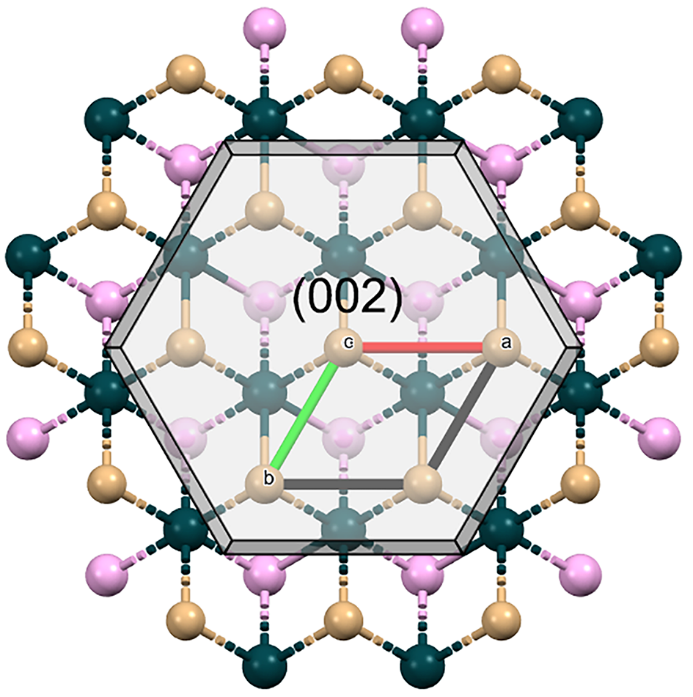
**

**Figure S8.** The calculated morphology of RhSeCl with face indexation.


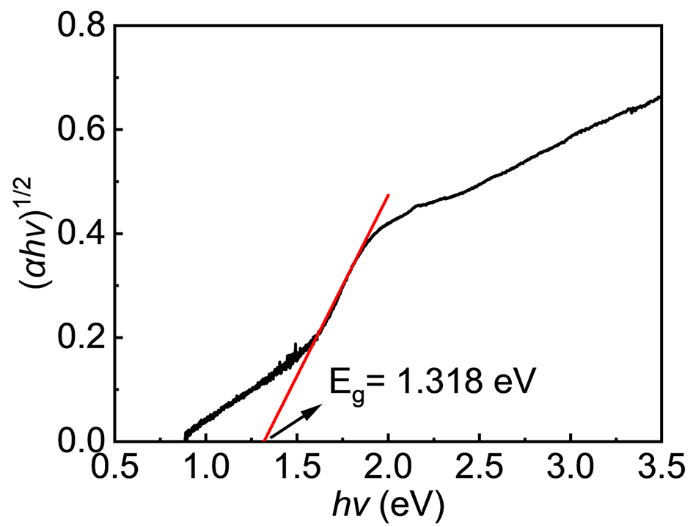


**Figure S9****.** Tauc plot for RhSeCl material collected using the diffuse reflectance method for indirect bandgap estimating.

To elucidate the optical characteristics of the RhSeCl sample, we meticulously performed absorption spectroscopy measurements on a SHIMADZU UV-2600 UV-Vis spectrophotometer to ascertain both the indirect and direct optical bandgaps. This calculation was based on the Tauc plot method derived from the UV-visible absorption spectrum, utilizing the equation: (*αhν*)=*A*(*hν*−*E_g_*_​_)*^n^*, where *α* is the absorption coefficient, *hν* is the photon energy, *E_g_*_​_ is the bandgap energy, *A* is a constant, and *n* takes a value of 2 for an indirect transition, as opposed to 1/2 for direct transitions. The indirect bandgap, which is more consistent with theoretical predictions that the material is an indirect bandgap semiconductor, was calculated to be around 1.318 eV. This providing a robust indication of the material's electronic structure and optical properties.


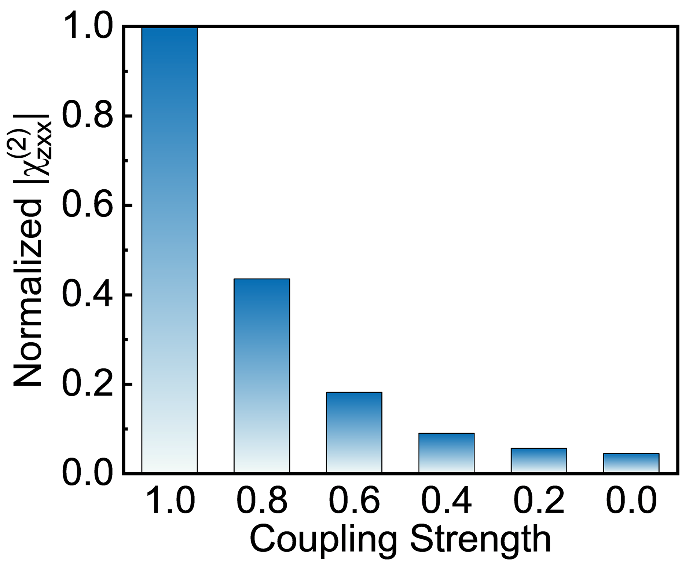


**Figure S10.** Intensity of peak A in RhSeCl with different coupling strength.


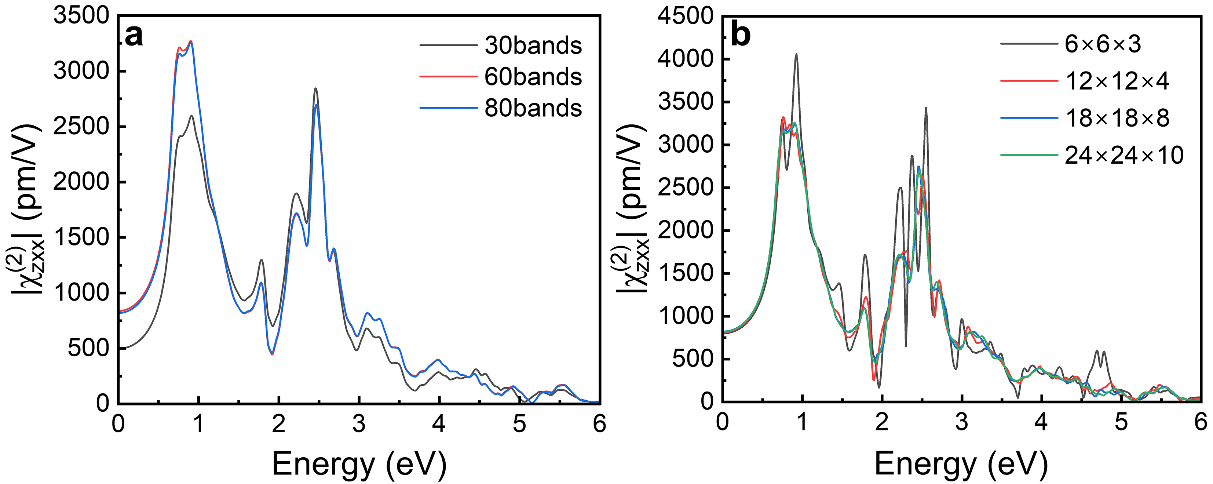


**Figure S11.** Convergence test of nonlinear optical response calculation. SHG susceptibility $\chi^{\left( 2 \right)}$ as a function of incident photon energy for (a) different bands and (b) k-points.

**Table S1.** Important crystal data and structure refinement parameters for RhSeCl at 30, 100 and 200 K.

| Formula | RhSeCl | | |
| --- | --- | --- | --- |
| fw / g·mol^–1^ | 217.32 | | |
| T / K | 30(2) | 100(2) | 200(2) |
| Radiation |  | Mo-Kα |  |
| Wavelength |  | 0.71073 Å |  |
| Space group, No. |  | *P*6_3_*mc* (No. 186) |  |
| Z |  | 2 |  |
| Cell dimensions |  |  |  |
| *a /* Å | 3.4874(4) | 3.4869(2) | 3.4874(2) |
| *c /* Å | 11.549(2) | 11.5428(7) | 11.5643(8) |
| *V /* Å^3^ | 121.64(4) | 121.540(16) | 121.802(16) |
| *ρ_calc_* / g·cm^−3^ | 5.934 | 5.938 | 5.926 |
| *μ_Mo Kα_* / mm^−1^ | 22.654 | 22.673 | 22.624 |
| GOF on *F*^2^ | 1.248 | 1.376 | 1.545 |
| *R*_1_ [*I* > 2*σ_(I)_*] *^a^* | 0.0325 | 0.0274 | 0.0518 |
| *wR*_2_ [*I* > 2*σ_(I)_*] *^a^* | 0.0666 | 0.0801 | 0.1617 |
| *R*_1_ [all data] *^a^* | 0.0422 | 0.0283 | 0.0524 |
| *wR*_2_ [all data] *^a^* | 0.0714 | 0.0807 | 0.1624 |
| *^a^ R*_1_ = ∑\|\|*F*_o_\| – \|*F*_c_\|\|/∑\|*F*_o_\|; *wR*_2_ = [∑[*w*(*F*_o_^2^ – *F*_c_^2^)^2^]/∑[*w*(F_o_^2^)^2^]]^1/2^, and *w* = 1/[*σ*^2^*F*_o_^2^ + (*A*·*P*)^2^ + *B*·*P*], *P* = (*F*_o_^2^ + 2*F*_c_^2^)/3; *A* and *B* are weight coefficients. | | | |

**Table S2.** Important crystal data and structure refinement parameters for RhSeCl at 300, 400 and 500 K.

| Formula | RhSeCl | | |
| --- | --- | --- | --- |
| fw / g·mol^–1^ | 217.32 | | |
| T / K | 300(2) | 400(2) | 500(2) |
| Radiation |  | Mo-Kα |  |
| Wavelength |  | 0.71073 Å |  |
| Space group, No. |  | *P*6_3_*mc* (No. 186) |  |
| Z |  | 2 |  |
| Cell dimensions |  |  |  |
| *a /* Å | 3.4901(2) | 3.4891(2) | 3.4919(3) |
| *c /* Å | 11.5941(7) | 11.6182(9) | 11.6525(12) |
| *V /* Å^3^ | 122.305(16) | 122.489(17) | 123.05(2) |
| *ρ_calc_* / g·cm^−3^ | 5.901 | 5.892 | 5.866 |
| *μ_Mo Kα_* / mm^−1^ | 22.531 | 22.497 | 22.395 |
| GOF on *F*^2^ | 1.298 | 1.362 | 1.192 |
| *R*_1_ [*I* > 2*σ_(I)_*] *^a^* | 0.0429 | 0.0351 | 0.0322 |
| *wR*_2_ [*I* > 2*σ_(I)_*] *^a^* | 0.1256 | 0.1112 | 0.0785 |
| *R*_1_ [all data] *^a^* | 0.0438 | 0.0363 | 0.0348 |
| *wR*_2_ [all data] *^a^* | 0.1274 | 0.1120 | 0.0800 |
| *^a^ R*_1_ = ∑\|\|*F*_o_\| – \|*F*_c_\|\|/∑\|*F*_o_\|; *wR*_2_ = [∑[*w*(*F*_o_^2^ – *F*_c_^2^)^2^]/∑[*w*(F_o_^2^)^2^]]^1/2^, and *w* = 1/[*σ*^2^*F*_o_^2^ + (*A*·*P*)^2^ + *B*·*P*], *P* = (*F*_o_^2^ + 2*F*_c_^2^)/3; *A* and *B* are weight coefficients. | | | |

**Table S3.** Refined atomic coordinates and isotropic displacement parameters for RhSeCl at 30, 100 and 200 K.

| Atoms | *Wyckoff* | *x* | *y* | *z* | *U_eq_ ^a^*(*Å^2^*) | *Occupancy* |
| --- | --- | --- | --- | --- | --- | --- |
| 30 K | | | | | | |
| Rh | *2b* | 2/3 | 1/3 | 0.49287(6) | 0.0064(4) | 1 |
| Se | *2b* | 1/3 | 2/3 | 0.37897(19) | 0.0060(6) | 1 |
| Cl | *2a* | 1.0 | 0 | 0.6220(5) | 0.0069(8) | 1 |
| 100 K | | | | | | |
| Rh | 2*b* | 2/3 | 1/3 | 0.50795(2) | 0.0032(4) | 1 |
| Se | 2*b* | 1/3 | 2/3 | 0.62127(19) | 0.0041(5) | 1 |
| Cl | 2*a* | 1.0 | 0 | 0.3787(5) | 0.0052(7) | 1 |
| 200 K | | | | | | |
| Rh | 2*b* | 1/3 | 2/3 | 0.50723(2) | 0.0060(17) | 1 |
| Se | 2*b* | 2/3 | 1/3 | 0.6201(5) | 0.0086(19) | 1 |
| Cl | 2*a* | 0 | 1.0 | 0.3761(11) | 0.010(3) | 1 |
| *^a^ U*_eq_ is defined as one-third of the trace of the orthogonalized *U*^ij^ tensor. | | | | | | |

**Table S4.** Refined atomic coordinates and isotropic displacement parameters for RhSeCl at 300, 400 and 500 K.

| Atoms | *Wyckoff* | *x* | *y* | *z* | *U_eq_ ^a^*(*Å^2^*) | *Occupancy* |
| --- | --- | --- | --- | --- | --- | --- |
| 300 K | | | | | | |
| Rh | 2*b* | 2/3 | 1/3 | 0.49110(2) | 0.0063(7) | 1 |
| Se | 2*b* | 1/3 | 2/3 | 0.3780(2) | 0.0077(8) | 1 |
| Cl | 2*a* | 1.0 | 0 | 0.6215(6) | 0.0094(11) | 1 |
| 400 K | | | | | | |
| Rh | 2*b* | 1/3 | 2/3 | 0.49169(2) | 0.0122(13) | 1 |
| Se | 2*b* | 2/3 | 1/3 | 0.3785(4) | 0.0138(15) | 1 |
| Cl | 2*a* | 0 | 1.0 | 0.6231(10) | 0.017(2) | 1 |
| 500 K | | | | | | |
| Rh | 2*b* | 2/3 | 1/3 | 0.49218(2) | 0.0143(7) | 1 |
| Se | 2*b* | 1/3 | 2/3 | 0.3794(3) | 0.0147(9) | 1 |
| Cl | 2*a* | 1.0 | 0 | 0.6210(7) | 0.0200(13 | 1 |
| *^a^ U*_eq_ is defined as one-third of the trace of the orthogonalized *U*^ij^ tensor. | | | | | | |

**Table S5.** Selected interatomic distances (Å) in RhSeCl from 30 to 500 K.

| Atoms pairs | Distances (Å) | Atoms pairs | Distances (Å) | Atoms pairs | Distances (Å) |
| --- | --- | --- | --- | --- | --- |
| 30 K | | 100 K | | 200 K | |
| Rh-Se×3 | 2.405(1) | Rh-Se×3 | 2.401(1) | Rh-Se×3 | 2.400(3) |
| Rh-Cl×3 | 2.506(3) | Rh-Cl×3 | 2.506(3) | Rh-Cl×3 | 2.521(8) |
| 300 K | | 400 K | | 500 K | |
| Rh-Se×3 | 2.404(1) | Rh-Se×3 | 2.406(3) | Rh-Se×3 | 2.406(1) |
| Rh-Cl×3 | 2.520(4) | Rh-Cl×3 | 2.528(7) | Rh-Cl×3 | 2.513(5) |

**Table S6.** Some Janus-related metal chalcohalides and their metal-halogen distances.

| compounds | metal-halogen distances (Å) | compounds | metal- halogen distances (Å) | compounds | metal- halogen distances (Å) | compounds | metal- halogen distances (Å) |
| --- | --- | --- | --- | --- | --- | --- | --- |
| RhCl_3_ | 2.30 | BiCl_3_ | 2.46-3.24 | BiBr_3_ | 2.80 | BiI_3_ | 2.91-2.97 |
| RhSeCl | 2.51 | BiSeCl | 3.19 | BiSeBr | 2.99 | BiSeI | 3.24-3.78 |
| RhTeCl | 2.57 | BiTeCl | 3.01 | BiTeBr | 3.01 | BiTeI | 3.07 |

**Table S7.** Fitting parameters of Rh K-edge EXAFS spectra of RhSeCl.

| Path | CN | R(Å) | σ^2 |  |
| --- | --- | --- | --- | --- |
| 1. Rh-Se | 3 | 2.41 | 0.004 | E0 = 23225.6 eV |
| 2. Rh-Cl | 3 | 2.53 | 0.01 | E0 shift: 0.59 eV |
| 3.Rh-Rh | 6 | 3.5 | 0.0062 | S0^2 = 0.9 |
| 4. Rh-Se | 3 | 4.23 | 0.01 |  |
| 5. Rh-Cl | 3 | 4.42 | 0.01 |  |
| 6.Rh-Se | 1 | 4.47 | 0.0059 |  |
| k-space range: from 2.6 to 14.0 Å^-1^ | | | | |

**Table S8.** Fitting parameters of Se K-edge EXAFS spectra of RhSeCl.

| Path | CN | R(Å) | σ^2 |  |
| --- | --- | --- | --- | --- |
| 1.Se-Rh | 3 | 2.41 | 0.004 | E0 = 12656.9 eV |
| 2.Se-Cl | 3 | 3.46 | 0.0091 | E0 shift: 6.21 eV |
| 3.Se-Se | 6 | 3.52 | 0.0094 | S0^2 = 0.76 |
| 4.Se-Cl | 3 | 3.6 | 0.004 |  |
| 5.Se-Rh | 3 | 4.33 | 0.01 |  |
| 6.Se-Rh | 1 | 4.49 | 0.004 |  |
| k-space range: from 2.9 to 14.0 Å^-1^ | | | | |

**Table S9.** The structure models of RhSeCl used in the EXAFS fitting.

| Path | R | CN | Path | R | CN |
| --- | --- | --- | --- | --- | --- |
| Rh-Se | 2.4 | 3 | Se-Rh | 2.4 | 3 |
| Rh-Cl | 2.51 | 3 | Se-Cl | 3.45 | 3 |
| Rh-Rh | 3.48 | 6 | Se-Se | 3.48 | 6 |
| Rh-Se | 4.23 | 3 | Se-Cl | 3.59 | 3 |
| Rh-Cl | 4.29 | 3 | Se-Rh | 4.23 | 3 |
| Rh-Se | 4.48 | 1 | Se-Rh | 4.48 | 1 |

**Table S10.** Fitting parameters of Rh K-edge EXAFS spectra of RhCl_3_.

| Path | CN | R(Å) | σ^2 |  |
| --- | --- | --- | --- | --- |
| 1.Rh-Cl | 6 | 2.34 | 0.0046 | E0 = 23227.6 eV |
| 2.Rh-Rh | 3 | 3.46 | 0.0086 | E0 shift: 0.46 eV |
|  |  |  |  | S0^2 = 0.9 |
| k-space range: from 2.6 to 14.0 Å^-1^ | | | | |

**Table S11.** Fitting parameters of Rh K-edge EXAFS spectra of Rh-foil.

| Path | CN | R(Å) | σ^2 |  |
| --- | --- | --- | --- | --- |
| 1.Rh-Rh | 12 | 2.69 | 0.0041 | E0 = 23220.6 eV |
| 2.Rh-Rh | 6 | 3.80 | 0.0071 | E0 shift: 4.10 eV |
|  |  |  |  | S0^2 = 0.88 |
| k-space range: from 2.6 to 14.0 Å^-1^ | | | | |

**Table S12.** Integrated COHP (ICOHP) up to the Fermi level for Rh/Bi - Se/Te and Rh/Bi - Cl/I in RhSeCl, RhTeCl, BiSeCl and BiTeI.

| RhSeCl | Rh-Se | Rh-Cl |
| --- | --- | --- |
| Bond length (Å) | 2.42 | 2.54 |
| ICOHP (eV) | -2.78 | -1.47 |
| RhTeCl | **Rh-Te** | **Rh-Cl** |
| Bond length (Å) | 2.57/2.59 | 2.53/2.58 |
| ICOHP (eV) | -2.56/-2.47 | -1.44/-1.30 |
| BiSeCl | **Bi-Se** | **Bi-Cl** |
| Bond length (Å) | 2.81/2.85 | 2.95/3.04 |
| ICOHP (eV) | -2.62/-2.36 | -1.13/-0.90 |
| BiTeI | **Bi-Te** | **Bi-I** |
| Bond length (Å) | 3.07 | 3.30 |
| ICOHP (eV) | -2.06 | -0.96 |
